# Fluorophenylalanine-Containing Dipeptides with Antibiofilm Activity Against *Pseudomonas aeruginosa* Discovered Through a Collaborative Undergraduate Research and Educational Program

**DOI:** 10.3390/molecules31132341

**Published:** 2026-07-03

**Authors:** Amy B. Dounay, Amelia A. Fuller, Dylan R. Y. Lawton, Sarah Buchman, Markus F. Bergstrom, Rose Gilmore, Emerson Hernly, Jake Hitchens, Justin Tee, Callista Tran, Nathan Weiler, Gregory G. Anderson, Olivia Hatton, Kathleen A. Marrs, Kristen Mudrack, Martin J. O’Donnell, Doug Schirch, Kevin Sullivan, J. Geno Samaritoni, William L. Scott

**Affiliations:** 1 Department of Chemistry and Biochemistry, Colorado College, 14 E. Cache La Poudre Street, Colorado Springs, CO 80903, USA; 2 Department of Chemistry and Biochemistry, Santa Clara University, 500 El Camino Real, Santa Clara, CA 95053, USA; 3 Department of Molecular Biology, Colorado College, 14 E. Cache La Poudre Street, Colorado Springs, CO 80903, USAohatton@coloradocollege.edu (O.H.); 4 Department of Biology, Indiana University Indianapolis, 723 W. Michigan Street, SL 330, Indianapolis, IN 46202, USAkmarrs@iu.edu (K.A.M.); 5 Department of Chemistry and Chemical Biology, Indiana University Indianapolis, 402 N. Blackford St., LD 326, Indianapolis, IN 46202, USAjsamarit@iu.edu (J.G.S.); wscott@iu.edu (W.L.S.); 6 Department of Chemistry, Milligan University, 1 Blowers Boulevard, Milligan, TN 37682, USA; 7 Department of Chemistry, Goshen College, 1700 South Main Street, Goshen, IN 46526, USA; dougms@goshen.edu; 8 Department of Chemistry, University of Indianapolis, 1400 East Hanna Avenue, Indianapolis, IN 46227, USA

**Keywords:** solid-phase peptide synthesis, prodrug, bioactive peptides, antimicrobial peptides, phenotypic screening, selective toxicity

## Abstract

Discovery of new antibiotic candidates is driven by urgent global health needs and evolving resistance to current treatments. In pursuit of novel peptide-based antibiotics, more than 100 dipeptides containing fluorinated phenylalanine (F-Phe) residues were synthesized by undergraduate researchers at eight institutions and evaluated against *Pseudomonas aeruginosa* (*P. aeruginosa*) in a primary biofilm formation assay. Inclusion of multistep synthesis experiments in curricular laboratory courses at different institutions prompted the prioritization of robust and adaptable procedures that were effective at generating large compound sets for study. Several dipeptides were identified that inhibited biofilm formation at concentrations of 1 μg/mL or less. Further study of two of them, (*S*,*S*)-4-F-Phe-Ala and (*S*,*S*)-3,4-diF-Phe-Ala, revealed two distinct biological profiles. Inhibition of biofilm formation by (*S*,*S*)-4-F-Phe-Ala was directly associated with growth inhibition. In contrast, (*S*,*S*)-3,4-diF-Phe-Ala did not significantly inhibit bacterial growth at concentrations up to 100 μg/mL. Mammalian cell lines treated with either dipeptide at much higher concentrations remained viable, demonstrating selective toxicity for *P. aeruginosa*. Taken together, these data indicate that dipeptides comprising F-Phe are promising leads for the discovery of new antibiofilm therapeutics.

## 1. Introduction

Persistent infections caused by *Pseudomonas aeruginosa* (*P. aeruginosa*) represent a major and escalating global health challenge due to their prevalence, clinical severity, and increasing resistance to existing antibiotics. The 2024 World Health Organization Bacterial Priority Pathogens List identified antibiotic-resistant *P. aeruginosa* among the top 15 pathogens for which new therapeutics are urgently needed, reflecting its particular concern in health-care settings worldwide [1]. Among patients with cystic fibrosis, *P. aeruginosa* is a predominant and difficult-to-treat pathogen contributing to progressive lung disease through long-term persistence in damaged airways [2]. Likewise, *P. aeruginosa* is a leading pathogen in ventilator-associated pneumonia, urinary tract infections, burn and wound infections, and systemic disease [3]. Notably, the incidence of multidrug-resistant *P. aeruginosa* infections continues to rise [4,5], underscoring the urgent need for new antimicrobial chemotypes. This need is further amplified by increasing global antibiotic consumption, which accelerates the evolution of resistance pathways [6]. Recent advances in understanding *P. aeruginosa* pathogenicity and virulence have prompted new strategies for therapeutic design, including antivirulence approaches that target inhibition of biofilm formation [7,8].

Unnatural dipeptides are attractive for their potential to inhibit *P. aeruginosa* functions selectively over mammalian cell functions. Microbial uptake of small peptides is mediated by multicomponent transport systems that supply essential nutrients and metabolic building blocks [9]. In vitro studies of di- and tripeptides containing L-3-fluorophenylalanine (L-3-F-Phe) have investigated their use as antifungal agents against *Candida albicans* [10]. Their antifungal activities are consistent with a prodrug hypothesis that these compounds are actively transported into the cell, where the 3-F-Phe is released and inhibits growth [10]. Similarly, selective cellular uptake of peptides and peptidomimetics into bacteria via ATP-binding cassette (ABC) transporters [11,12] may be leveraged for antibacterial drug design: di- and oligopeptides may enter via these pathways and disrupt cellular processes, including planktonic bacterial growth and biofilm formation. Exploiting bacterial transport machinery to deliver and release active compounds may enable both new discovery and repurposing of previously reported antibiotics. For example, fluoro-substituted phenylalanines (F-Phe), which have exhibited toxicity in rodent models [13,14], may be delivered as short peptides. Previous studies demonstrated that dipeptides with 4-fluorophenylalanine can infiltrate *P. aeruginosa* and inhibit biofilm formation [15]. Dipeptides that include F-Phe have also shown promising antifungal activity against *Candida albicans* and *Cryptococcus neoformans* [15,16,17]. Systematic evaluation of F-Phe dipeptides will clarify their ability to selectively disrupt essential functions in *P. aeruginosa* over mammalian cells.

**Figure 1:**
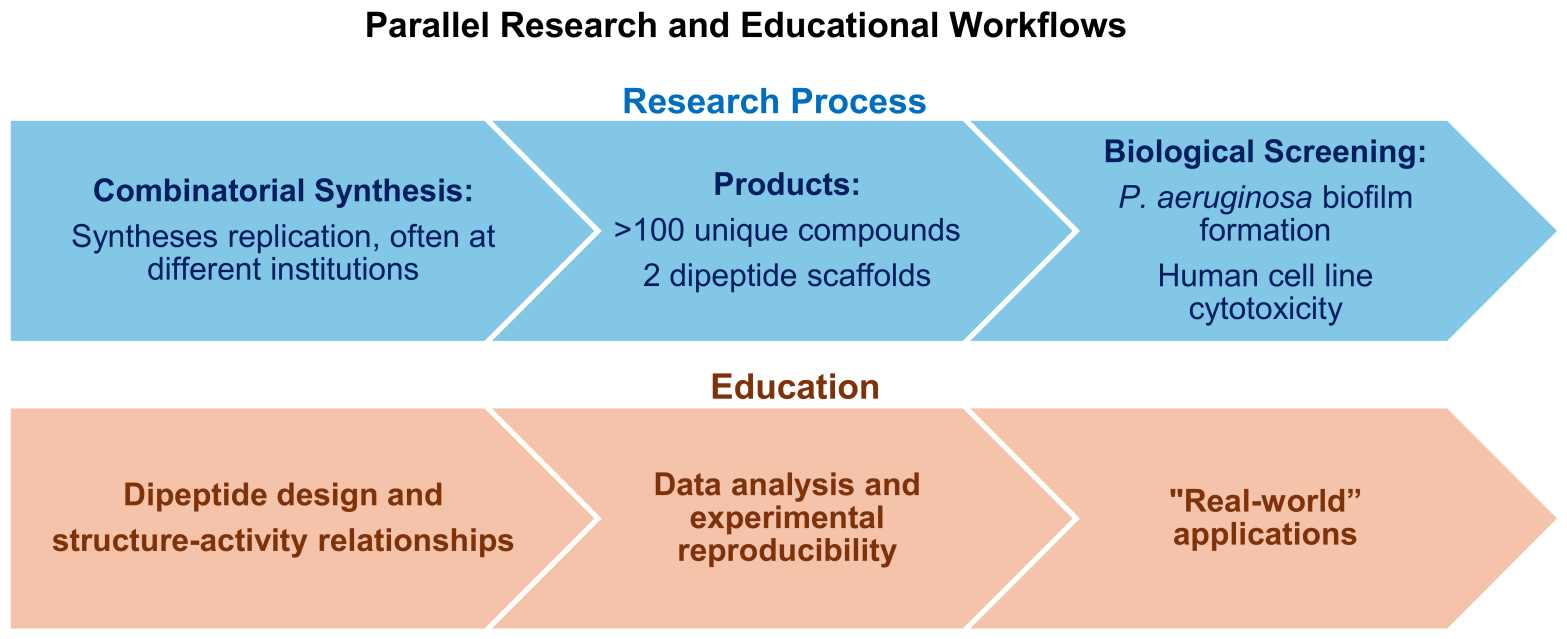
Schematic illustration of the parallel research process (blue) and education (tan) workflows in the D3 program.

Academic laboratories can advance the discovery of next-generation therapeutics against *P. aeruginosa* by identifying novel drug scaffolds, generating analog libraries for structure–activity studies, and evaluating biological activity. We have previously reported on the advantages of a distributed drug discovery (D3) approach to meet the significant challenge of synthesis and evaluation of libraries of diverse compounds, while also advancing educational objectives (Figure 1) [18,19,20,21]. Following the D3 model, which shares similarities with other multi-institutional drug discovery initiatives [22,23,24,25], large numbers of undergraduate students enrolled in laboratory courses are deputized for both synthesis and biological testing. This has been a successful approach for identifying compounds with antimicrobial and antiparasitic activities [16,23]. The engagement of many sets of hands is advantageous for advancing drug discovery research objectives; simultaneously, the inclusion of “real-world” contextualized experiences in laboratory curricula enhances student learning [21]. We and others have reported on the value of course-based undergraduate research experiences (CUREs) to promote student learning, increase intrinsic motivation in STEM, and improve student retention, including that of historically marginalized groups [26]. Because CUREs are implemented within the normal laboratory course, they lower barriers to undergraduate research access while providing many of the same benefits [26,27].

Unnatural dipeptides are attractive targets for study by undergraduate students participating in this work through CUREs or mentored research. First, the modularity of dipeptides provides an approachable context to introduce students to basic medicinal chemistry concepts of structure-activity relationships [21,28,29]. The synthesis of peptides on solid support utilizes well-documented reactions [30] suitable for novice chemists. Because the reactions are reliable, they can be readily adapted to different laboratory environments, enabling the contributions of collaborators at different sites with different time and resource constraints in the curricular or research laboratory (Table S1). Beyond synthesis, preliminary biological evaluation of unnatural dipeptides’ effects on biofilm formation can be executed in mentored or curricular laboratory environments (vide infra). Thus, the D3 program is adaptable to engage undergraduate students with different experiments applied to early drug discovery. Lastly, involving students in either the replicated synthesis or biological screening of unnatural dipeptides underscores the importance of experimental reproducibility.

**Figure 2:**
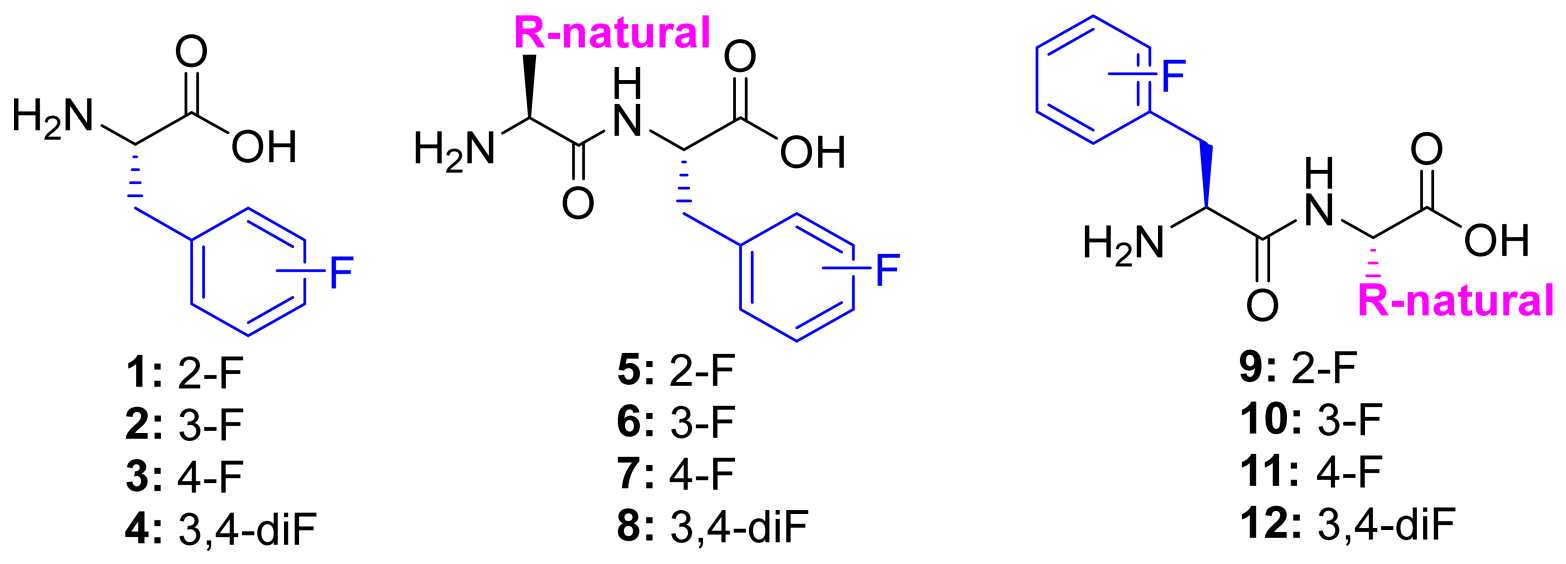
General structures of (*S*)-fluorophenylalanines (F-Phe, **1**–**4**), C-terminal (**5**–**8**) and N-terminal (**9**–**12**) (*S*)-F-Phe dipeptides; R = Natural amino acid side chain (magenta). The F-Phe side chain residue is shown in blue.

This study describes the synthesis and evaluation of unnatural dipeptides incorporating fluoro- or difluoro-substituted phenylalanine (Figure 2) for inhibition of *P. aeruginosa* biofilm formation. Three distinct F-Phe residues (**1**–**3**) and one di-F-Phe residue (**4**) were selected based on previously reported biofilm inhibition activities of halogenated phenylalanines [15] (Figure S2). Since 2016, robust and adaptable synthesis procedures have been applied by undergraduate students at eight different institutions to prepare more than 100 unique unnatural dipeptides (Table S1). Undergraduate students also screened analogs in microbiology laboratory courses and in mentored research settings, using a phenotypic assay to assess dipeptide inhibition of *P. aeruginosa* biofilm formation. Trends in these data revealed how dipeptide stereochemistry and sidechain structure impact biofilm formation. Two unnatural dipeptides, **11b** and **12b** (R = Me) and their respective (*S*)-F-Phe residues, **3** and **4**, were further evaluated to discern their effects on bacterial growth, biofilm formation, and toxicity to mammalian cell lines. Encouragingly, we have demonstrated that both **11b** and **12b** selectively inhibit *P. aeruginosa* cellular functions at concentrations as low as 1 μg/mL, but do not exhibit cytotoxicity in mammalian cells at these concentrations.

## 2. Results

### 2.1. Synthesis of Unnatural Dipeptides

The synthetic unnatural dipeptide targets **5**–**12** were designed to explore how residue sidechains, sequence, and stereochemistry influence inhibition of *P. aeruginosa* biofilm formation. We anticipated that the hypothesized cellular uptake of dipeptides, hydrolysis of the peptide bond, and/or other biochemical mechanisms that influence cellular functions may be sensitive to these variables. Three distinct F-Phe residues (**1**–**3**) and one diF-Phe residue (**4**) were incorporated to discern the effects of fluorine position on biological activities. These residues were chosen based on the observed activities of racemic versions of **1**–**4** to inhibit biofilm formation (Figure S2). The second position of the dipeptide was a natural amino acid residue, and the sequence was varied by including the natural residue either at the N-terminus (**5**–**8**) or the C-terminus (**9**–1**2**). For **5**–**8**, analogs that included racemic F-Phe or diF-Phe residues were also prepared to scrutinize the effects of stereochemical configurations on the dipeptides’ biological activities.

Solid-phase synthesis of the unnatural dipeptides was executed by students at eight institutions collaborating through the D3 program, either as part of their curricular laboratory work or while engaged in faculty-mentored research (Table S1). Compound syntheses were replicated both within a site and, frequently, across sites to ensure procedures were robust and amenable to different laboratory environments. To introduce students to the concepts of parallel combinatorial synthesis, the commercially available “Bill-Board” apparatus [31] was used at most sites (Figure S1). The Bill-Board arrays six solid-phase synthesis reaction vessels in a 3 × 2 grid layout. An example synthetic array is provided in Figure S1B. Alternatively, some laboratories elected to use traditional solid-phase chemistry synthesis vessels or commercially available polypropylene syringes equipped with a fritted filter to carry out individual reactions [32].

**Scheme 1:**
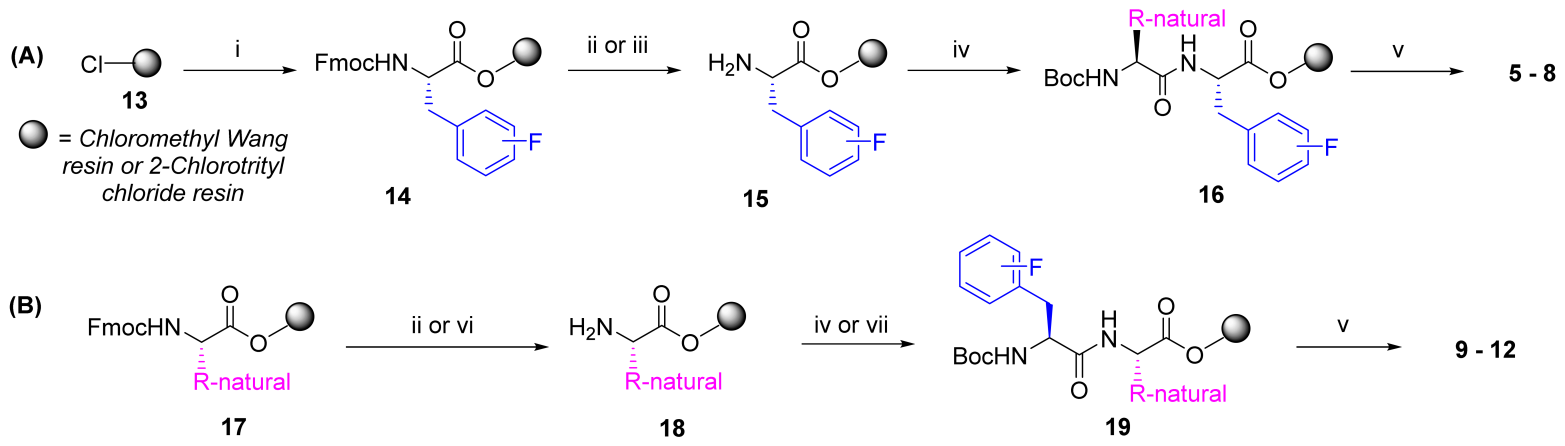
Synthesis of F-Phenylalanyl Dipeptides (**A**) Synthesis of dipeptides **5**–**8**; (**B**) Synthesis of dipeptides **9**–**12**. R = Natural amino acid side chain (magenta). The F-Phe side chain residue is shown in blue. Reagents and conditions: (i) Fmoc-F-Phe-OH (6.0 equiv), KI (0.6 equiv), DIPEA (6.0 equiv), NMP, rt; (ii) 20% Piperidine in DMF or NMP; (iii) 30% 4-methylpiperidine in DMF or NMP; (iv) Boc-AA-OH (5 equiv), DIC (5 equiv), HOBt (5 equiv), DMF or NMP; (v) TFA/DCM/H_2_O (35:60:5); (vi) 20% 4-methylpiperidine in ethyl acetate; (vii) Boc-AA-OH (3.4 equiv), T3P (6.8 equiv), DIPEA (3.4 equiv), ethyl acetate; DMF: *N*,*N*-dimethylformamide; NMP: *N*-methyl-2-pyrrolidone; AA: amino acid; DIC: *N*,*N*′-diisopropylcarbonyldiimide; HOBt: 1-hydroxybenzotriazole hydrate; TFA: trifluoroacetic acid; DCM: dichloromethane; T3P: propylphosphonic anhydride; DIPEA: *N*,*N*-diisopropylethylamine.

Early synthetic efforts in the D3 program prepared unnatural dipeptide analogs with C-terminal F-Phe and diF-Phe residues (**5**–**8**, Scheme 1A). The initial synthetic route involved the alkylation of the benzophenone imine of glycine-Wang and produced dipeptides with racemic F-Phe or di-F-Phe *C*-termini (Scheme S2) [19]. Later, the enantiopure Fmoc-F-Phe or di-F-Phe residues were directly loaded onto the resin (**14**) by the laboratory instructor or staff. Fmoc removal then afforded **15**. Fifteen *N*-Boc-protected natural amino acids were subsequently coupled to resin-bound residue **15** using DIC/HOBt-mediated acylation reactions. Acidic cleavage from the resin with concomitant protecting group removal provided the dipeptides (**5**–**8**). As summarized in the Supporting Information (Table S2), these methods yielded access to a large set of unnatural dipeptides **5**–**8** in crude yields of 25–97%, many of which were prepared in replicate by different students.

Recent D3 laboratory curricula have focused on synthesizing unnatural dipeptides bearing N-terminal 2-F-Phe (**9**), 3-F-Phe (**10**), 4-F-Phe (**11**), or 3,4-diF-Phe (**12**) residues and canonical amino acids at the C-terminus (Scheme 1B). Student researchers removed the Fmoc protecting group from **17**, then acylated the amine (**18**) with the Boc-protected 2-F-Phe, 3-F-Phe, 4-F-Phe or 3,4-diF-Phe to afford **19**. The coupling step was initially completed under the “traditional” conditions using DIC/HOBt and DMF or NMP as solvent. More recently, peptide couplings have been achieved under “greener” conditions using T3P^®^ (Sigma-Aldrich, St. Louis, MO, USA) with DIPEA and ethyl acetate as solvent [33]. Cleavage from the resin and protecting group removal provided the dipeptides (**9**–**12**).

Considering the 20 canonical amino acids and four F-Phe/diF-Phe analogs, 80 unnatural dipeptides with the general structure F-Phe/diF-Phe–canonical amino acid (**9**–**12**) were possible. Following the approach outlined in Scheme 1B, 60 dipeptides were synthesized across five sites: none of the dipeptides contained tryptophan or cysteine, and only two contained aspartic acid or histidine, and only one contained asparagine. These residues were excluded to avoid complications with additional protecting groups and for ease of chromatographic isolation of final dipeptide products. The robustness of the synthetic procedures was demonstrated by replication of 18 syntheses, including 11 performed independently by student researchers at more than one site (Table S3).

### 2.2. Biological Evaluation of Unnatural Dipeptides

#### 2.2.1. Inhibition of *P. aeruginosa* Biofilm Formation by Unnatural Dipeptides

A phenotypic biofilm formation assay was selected as a primary screen to assess the effect of the unnatural dipeptides on *P. aeruginosa*. Because phenotypic screening strategies identify bioactive compounds based on functional outcomes, this approach can enable discovery of new chemotypes and avoid biasing compound selection based on known mechanisms of action [34]. Briefly, *P. aeruginosa* cultures were treated with dipeptides at concentrations ranging from 0.5 μg/mL to 20 μg/mL, and biofilms were quantified using a crystal violet assay [35], which was adaptable to the undergraduate instructional laboratory environment. For these preliminary studies, the PA14 laboratory strain of *P. aeruginosa* [36], one of the most frequently used strains for assessing novel therapeutics, was selected. A curricular module was installed into an undergraduate biology laboratory course to involve students in testing crude dipeptides **5**–**8** (purity measurements typically >85%) prepared in organic chemistry laboratory courses for their impact on *P. aeruginosa* biofilm formation.

Several important findings emerged from the evaluation of unnatural dipeptides **5**–**8** in curricular laboratories. Preliminary screens used concentrations of 0.5–20 µg/mL, and few peptides markedly reduced biofilm formation at <5 µg/mL. Therefore, for initial structure–activity relationship (SAR) evaluation, all compounds were screened against *P. aeruginosa* at a single concentration of 1 µg/mL in the primary biofilm inhibition assay (Table S2). First, the effect of chirality at the C-terminal residue was evaluated by comparing dipeptide scaffold variants **5**–**8** that included an N-terminal alanine and (*R*)-F-Phe or (*R*)-3,4-diF-Phe, (*S*)-F-Phe or (*S*)-3,4-diF-Phe, or racemic (*RS*)-F-Phe or (*RS*)-3,4-diF-Phe residues. The dipeptides containing (*S*)-F-Phe or (*S*)-diF-Phe residues, which match the chirality of natural L-Phe, were significantly more active than dipeptides with the (*R*)- or (*RS*)-F-Phe or -3,4-diF-Phe residues (Table S2). Next, out of 67 dipeptides screened, 13 compounds (19%) reduced biofilm formation by at least 50% relative to the control (Table S2). The most potent compound in this screen, (*S*,*S*)-Ala-3,4-diF-Phe (**8b**), reduced biofilm formation to 19% relative to control (Table S2); its sequence isomer, **12b**, was studied in more detail (vide infra). Although the integration of biofilm formation measurements into classroom experiments enabled the identification of active compounds and the relationship between residue stereochemistry and observed biofilm inhibition, reproducibility of the data in the classroom setting was highly variable between student experimentalists and between compound lots with varied purities.

Building on these initial data, 59 unique unnatural dipeptides **9**–**12** containing N-terminal (*S*)-F-Phe or (*S*)-di-F-Phe were prepared and purified. They were screened in mentored research laboratories in the *P. aeruginosa* biofilm formation assay at a concentration of 1 μg/mL (Table 1 and Table S3). No dipeptides containing 3-F-Phe (**10**) reduced biofilm formation below 50% of control at this concentration. Only one dipeptide containing 2-F-Phe (**9c**) reached this activity threshold, though several approached 50%. The five dipeptides with the highest inhibition of biofilm formation at this concentration—**11a**, **11b**, **11c**, **12a**, and **12d**—featured an N-terminal 4-F-Phe or 3,4-diF-Phe and an aliphatic C-terminal residue (e.g., Gly, Ala, Val, Leu). The preliminary biological screens were replicated on at least two compound lots for 14 of the compounds evaluated; these lots were prepared by different individual researchers, often at different institutions (Table S3).

#### 2.2.2. Bacterial Growth vs. Biofilm Formation by Unnatural Dipeptides **11b** and **12b**

**Figure 3:**
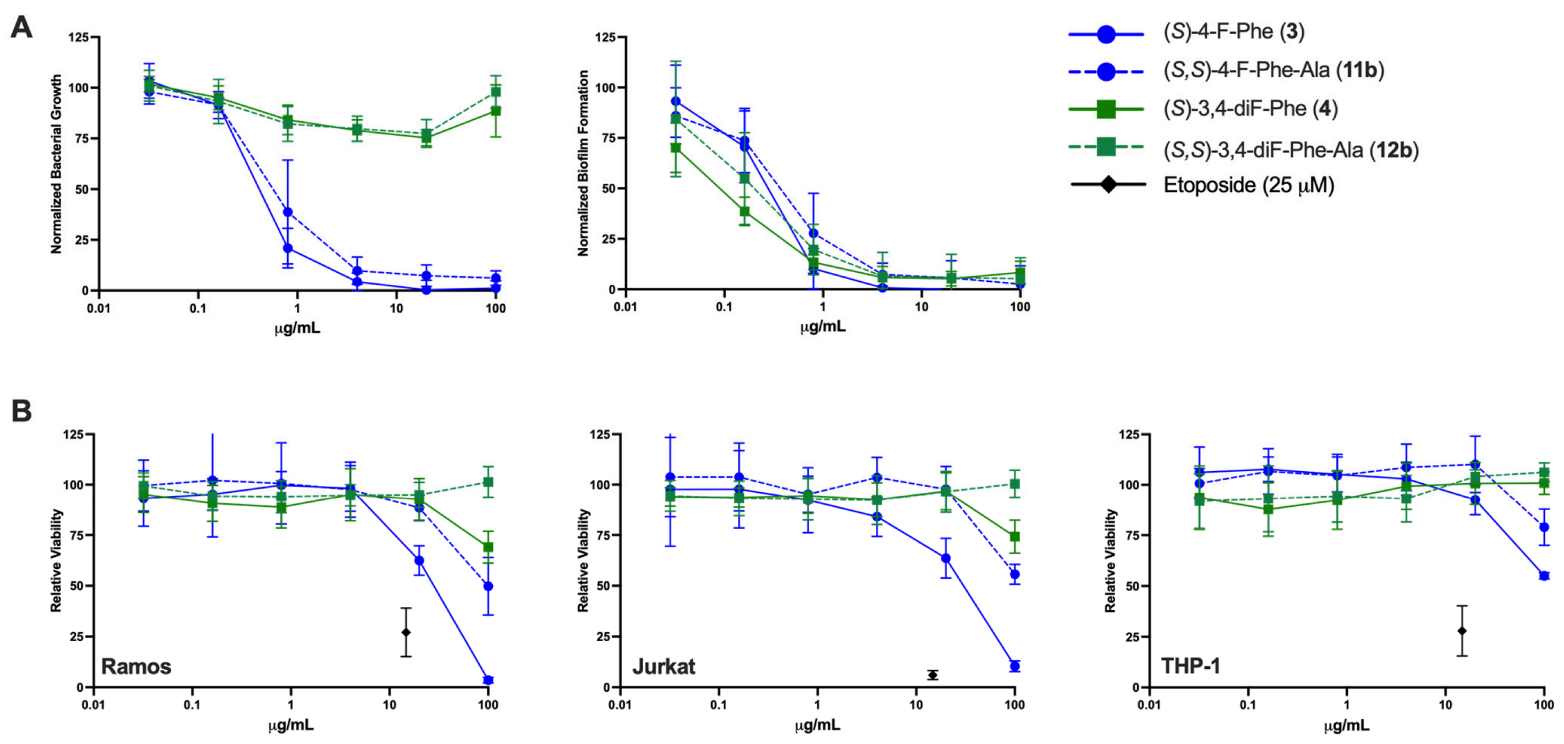
Unnatural dipeptides **11b** and **12b** dose–response assays. (**A**) *P. aeruginosa* cultures were treated as shown or with tobramycin (10 μg/mL) as a positive control. After 24 h, planktonic bacterial growth (left panel) was measured prior to assessing biofilm formation (right panel) following methods reported in Section 4.2, below. For both bacterial growth and biofilm formation, samples were normalized to untreated bacteria and media with vehicle-only samples. For each treatment, averages of 5–6 biological replicates testing a single compound lot are shown, and error bars represent standard deviation. (**B**) Ramos (left panel), Jurkat (middle panel), and THP-1 (right panel) cells were treated as shown or with 25 μM etoposide as a positive control. Samples were normalized to vehicle-treated cells and vehicle-treated media. After 48 h, viability was measured using a standard MTS assay. For each treatment, averages of 5–6 biological replicates testing the same compound lot in A are shown, and error bars represent standard deviation.

To further probe the biological effects of these unnatural dipeptides on *P. aeruginosa*, bacterial growth inhibition and biofilm formation were evaluated for two structurally related compounds, **11b** ((*S*,*S*)-4-F-Phe-Ala) and **12b** ((*S*,*S*)-3,4-di-F-Phe-Ala) (Figure 3 and Table 2). As the most potent inhibitor of biofilm formation, **11b** was selected for more in-depth evaluation. Dipeptide **12b** was selected for direct comparison to **11b** to probe the effect of the additional fluorine substitution. Because these dipeptides are putative prodrugs, their activities were compared with those of the corresponding amino acids, **3** (4-F-Phe) and **4** (3,4-diF-Phe), the respective presumed active species. All four compounds were evaluated in six-point dose–response assays using five-fold serial dilutions to determine IC_50_ and IC_90_ values for *P. aeruginosa* bacterial growth and biofilm formation (Figure 3A). IC_50_ and IC_90_ values are useful metrics in early-stage preclinical discovery and may be less variable than minimum inhibitory concentration (MIC) values [37,38,39]. Conversely, MIC and minimum biofilm inhibitory concentration (MBIC) values, though widely reported in antibiotic research, are more relevant to clinical applications. Each dipeptide exhibited activity comparable to its parent fluorinated amino acid. However, mono- and difluorinated analogs displayed distinct profiles. The monofluorinated compounds (**11b** and **3**) inhibited both bacterial growth and biofilm formation, with IC_50_ values of 0.57 and 0.42 μg/mL for growth inhibition and 0.36 and 0.26 μg/mL for biofilm inhibition, respectively. In contrast, the difluorinated compounds (**12b** and **4**) inhibited biofilm formation (IC_50_ = 0.17 and 0.08 μg/mL, respectively) but showed no measurable inhibition of bacterial growth (Figure 3A and Table 2). The low (<3 μg/mL) IC_90_ values for both **11b** and **12b** likewise confirm that these dipeptides have potent impacts on biofilm formation. Modest shifts in measurements from the preliminary single-point assays (Table S3) to the dose–response assays may be attributed to minor differences in assay format and increased number of replicates for the latter data sets.

#### 2.2.3. Selective Toxicity of **11b** and **12b** for *P. aeruginosa* and Mammalian Cells

To explore the selectivity of their biological activities, the cytotoxicity of **11b** and **12b** and their corresponding amino acids was assessed against human Ramos, Jurkat, and THP-1 cells. A six-point dose–response assay using 10-fold serial dilutions was used to determine their CC_50_ values, using etoposide as a positive control [40,41,42] (Figure 3B and Table 2). Compounds **4**, **11b**, and **12b** showed limited cytotoxicity (≤50%) at concentrations up to 100 μg/mL; compound **3** showed weak toxicity against Ramos cells with a CC_50_ value of 22.1 μg/mL. A selectivity index (SI) was calculated as the ratio of the CC_50_ in the most sensitive human cell line to the *P. aeruginosa* growth IC_50_. The selectivity index for the putative prodrug **11b** (SI > 175) was significantly higher than its parent F-Phe (**3**) (SI = 53). SI values were not calculated for **4** and **12b** because neither compound inhibited Ramos viability nor *P. aeruginosa* growth at the highest concentrations tested. The high selectivity overall reflects the significant sensitivity of both compounds toward *P. aeruginosa* relative to human cells.

## 3. Discussion

### 3.1. Antibiofilm Activity of Unnatural Dipeptides

The phenotypic *P. aeruginosa* assay screening of dipeptides from both isomeric libraries, **5**–**8** and **9**–**12**, identified dipeptides with promising antibiofilm activity. This screening also revealed preliminary SAR trends associated with fluorine substitution, the identity and stereochemistry of the natural amino acid residue, and the residue sequence. In the initial screen of scaffolds **5**–**8** at 1 μg/mL, analogs of **7** and **8**, containing 4-F-Phe or 3,4-diF-Phe residues, respectively, were most common among the most active biofilm inhibitors. Additionally, dipeptides with relatively small, hydrophobic natural amino acid residues (e.g., Ala, Val, Thr) showed the most potent inhibition of biofilm formation in this dataset. Similar trends were observed in the preliminary evaluation of the sequence isomer dipeptides (i.e., scaffolds **9**–**12**, containing the F-Phe residues at the N-terminus, Table 1 and Table S3). In this series, no dipeptides containing 3-F-Phe (**10**) reduced biofilm formation below 50% of control at 1 μg/mL, and only one dipeptide containing 2-F-Phe (**9c**) exceeded 50% inhibition of biofilm formation. The five dipeptides with the lowest biofilm formation at this concentration—**11a**, **11b**, **11c**, **12a**, and **12d**—featured an N-terminal 4-F-Phe or 3,4-diF-Phe and a C-terminal aliphatic residue (e.g., Gly, Ala, Val, Leu). Taken together, these preliminary screening results highlighted key structural features associated with potent antibiofilm activity.

Interestingly, SAR trends related to amino acid stereochemistry were consistent with our initial prodrug hypothesis, although they do not confirm this mechanism of action. One key finding from the initial biofilm inhibition screening of **5**–**8** was that the stereochemistry of the fluoro- or difluorophenylalanine residue was important for the measured dipeptide activity. (*S*)-F-Phe or (*S*)-diF-Phe chirality, which matches the chirality of natural L-Phe, was important for potent inhibition of biofilm formation (Table S2). This finding is consistent with the proposed prodrug hypothesis: we expect that the stereochemical configuration is important for transporters’ recognition of dipeptides for active transport into bacterial cells and/or for enzyme-mediated hydrolysis of the dipeptide. These promising preliminary results prompt additional experiments that will explore the origin of the observed trends.

The most potent inhibitor of biofilm formation, **11b**, was selected for follow-up studies, including dose–response assays. For direct comparison of the effect of difluoro-substitution of the N-terminal residue, **12b** was also selected for dose–response assays. The *P. aeruginosa* dose–response assays revealed a strikingly different profile for difluorinated dipeptide **12b** compared to the monofluorinated **11b**, suggesting that the structurally similar dipeptides may have different modes of action. The potency of dipeptide **12b** in the biofilm formation assay (IC_50_: 0.17 μg/mL) was comparable to that of **11b** (IC_50_: 0.36 μg/mL); however, **12b** did not inhibit planktonic bacterial growth at concentrations up to 100 μg/mL (IC_50_ > 100 μg/mL), whereas **11b** did (IC_50_: 0.57 μg/mL). These data suggest that the biofilm inhibitory activity of **11b** is likely due to the inhibition of bacterial growth, whereas a different mechanism of action may be responsible for the biofilm inhibition activity of **12b**.

The distinctive profile of dipeptide **12b** warrants further study to assess its potential as a “pathoblocker,” or antivirulence agent. Recent reviews have highlighted the advantages of drugs that reduce bacterial pathogenicity (e.g., by inhibiting biofilm formation) rather than killing bacteria or impairing viability, as traditional antibiotics do [7,43]. Because antivirulence agents do not kill bacteria, they may impose lower selection pressure for resistance, slowing the emergence of drug-resistant strains [7,43]. These agents instead disrupt host–pathogen interactions, potentially reducing side effects, including microbiome disruption, while allowing the host immune system to clear the infection [43]. Although some antivirulence agents may be effective as monotherapy, they may be especially valuable as adjuvants co-administered with antibiotics against highly resistant pathogens. For example, *P. aeruginosa* virulence blockers that act synergistically in combination with antibiotics, such as tobramycin and gentamycin, have been reported [44,45,46].

The similar profiles of the biological activities of **11b** and **12b** with their corresponding amino acids (**3** and **4**, respectively) also prompt future studies to evaluate our initial prodrug hypothesis (Figure 3, Table 2). The measured IC_50_ values for **11b** and **3** were similar for both inhibition of bacterial growth (**11b**: IC_50_ = 0.57 μg/mL; **3**: IC_50_ = 0.42 μg/mL) and for biofilm formation (**11b**: IC_50_: 0.36 μg/mL; **3**: IC_50_: 0.26 μg/mL). Likewise, the bioactivity of amino acid **4** tracked with its corresponding dipeptide (**12b**). Neither inhibited bacterial growth at the concentrations evaluated, and both had comparable measured IC_50_ values for inhibition of biofilm formation (**12b**: IC_50_: 0.17 μg/mL; **4**: IC_50_: 0.08 μg/mL). Although similar trends in these data again supported the hypothesis that **12b** is a prodrug of **4**, further studies will be directed at exploring the specific mechanism of action. These will include time-course studies to quantify release of **3** or **4** upon incubation of *P. aeruginosa* with **11b** or **12b**, respectively, to verify whether the dipeptides are serving as prodrugs.

A series of experiments is planned to further characterize the effects of our most promising compounds, including **11b** and **12b**, against *P. aeruginosa*. First, these compounds will be evaluated using a wider panel of *P. aeruginosa* strains, including clinical isolates. Additionally, the dipeptides’ potential utility as antivirulence agents will be assessed by determination of MBIC values. The dipeptides’ potential as adjuvants will be evaluated in co-dosing studies with known antibiotics (e.g., gentamycin or tobramycin). Furthermore, their effectiveness at eradicating pre-formed biofilms will be quantified by determination of minimum biofilm eradication concentrations (MBEC). Additional microscopy experiments, such as scanning electron microscopy (SEM) and confocal laser scanning microscopy (CLSM), may be used to visualize the effects of dipeptides identified here on biofilm architecture. Finally, the bactericidal properties of these compounds will be evaluated through time-kill kinetics experiments and by determination of minimum bactericidal concentrations (MBC).

Notably, neither **11b** nor **12b** showed significant cytotoxic effects against the three human cell lines (Ramos, Jurkat, and THP-1, Figure 3B, Table 2) in concentrations up to 100 μg/mL. The selective activities of **11b** and **12b** toward *P. aeruginosa* over human cells support further exploration of the specific molecular targets or the mechanisms of action for inhibition of *P. aeruginosa* growth and biofilm formation by **11b** and **12b** [47]. Future studies will interrogate whether or not **11b** or **12b** can be internalized into human cells; findings from these will also help us understand the origin of the selective activities. If the dipeptides are indeed serving as prodrugs, this strategy could alleviate toxicity liabilities reported for the active F-Phe components, such as **3** [13,14]. The demonstrated in vitro selectivity of the compounds for *P. aeruginosa* over mammalian cells additionally motivates studies to explore whether the biological activities observed for **11b** and **12b** are specific to this pathogen or if the dipeptides interact with other microorganisms, including other bacterial species.

### 3.2. Collaborative Undergraduate Research and Education

Centering undergraduate students in the synthesis and functional study of unnatural dipeptides reinforced the importance of designing reproducible and adaptable procedures that can be adopted at institutions with varied environments. In the synthesis of dipeptides **9**–**12**, there is considerable flexibility in the experiment timing, equipment, and specific reagents and solvents used. For example, when executing synthesis within curricular labs at different institutions, the scheduled duration and frequency of the course meetings dictated where in the synthesis and for how long syntheses may need to be paused. Across the varied laboratory environments, different personnel (i.e., students with varied levels of synthesis experience) may dictate the choice of synthesis equipment. The institutions engaged here have used different equipment with equal success to prepare the many compounds studied. Further, we have demonstrated that a range of different reagents and solvents can be employed to accomplish most of the synthetic steps, as detailed in the parallel procedures in the Supporting Information. A final consideration for inclusion of synthesis experiments in curricular laboratories was the availability of instructors and/or support personnel. Custom resins used in the synthesis of **5**–**8** were often prepared by laboratory instructors or support staff, whereas the synthesis of **9**–**12** used commercially available Wang resins pre-loaded with Fmoc-protected natural amino acids (**17**). We found the latter was advantageous to limit the demands on instructional staff. Altogether, the successful synthesis of many compounds at varied institutions confirmed that synthesis protocols were compatible with a wide range of laboratory schedules and formats across institutions.

Undergraduate students engaged with D3 research—both in synthesis and in biological evaluation of dipeptides—through curricular laboratory courses and/or by working alongside faculty in mentored student research experiences (Table S1). Students in these different environments often have varying levels of experience, motivation for, and time to allocate to experimental work. Through our experiences, we have found that synthesis and biological evaluation campaigns undertaken by students in curricular laboratory courses are most useful for initial screening. The data sets generated by many students working in parallel can be used to identify trends in synthesis outcomes and/or in biological effects, as described in this work. For example, certain dipeptides, such as those containing Cys, were reproducibly challenging to isolate across locations, whereas others were produced more consistently in high yields across sites. With respect to the biological effects of dipeptides, the influence of the stereochemical configuration of the F-Phe or diF-Phe residue was apparent and reproducible. Nonetheless, the data tend to have large variability from student to student, which parallels the heterogeneity of students’ engagement with and skill in their experimental work. As such, we have relied on students working in mentored research experiences to generate more reliably high-quality data that is suitable for publication. In this work, we have emphasized data obtained by mentored research students (Table 1 and Table 2, Figure 3).

Importantly, students involved in D3 projects in all environments were introduced to the ideas of experiment replication in several ways. In curricular laboratories, multiple students or teams were assigned to prepare or test the biological activity of a given compound, enabling students to compare their results (crude purities, yields and/or biofilm inhibition) with others. In the synthesis laboratories, student teams were frequently assigned to prepare a “control” dipeptide, a compound that was prepared by all students in the course. Comparing synthetic results of “control” compound yields and crude purities allowed laboratory leaders to validate reproducibility of the protocols. Similarly, positive and negative controls were included in the biofilm inhibition experiments to validate students’ technical skills and ensure data reliability.

## 4. Materials and Methods

### 4.1. Synthesis of Dipeptides

As noted in the text, different institutions used varied procedures to match their resources and environment. Synthesis reagents and solvents were ordered from different vendors by different institutions, and representative vendors are included in the methods described below. Commonly used synthetic methods are reported here, and variations on these are described in full in the Supporting Information.

#### 4.1.1. Synthesis of Dipeptides **5**–**8**

The synthesis methods used to prepare dipeptides **5**–**8** have been previously reported [16], and methods are described in full in the Supporting Information. The following compounds have been previously reported and characterized: Table S2, entries 1, 3, 5, 13, 16, and 45 [15]. Purity and identity of all synthetic dipeptides were confirmed by LC-MS. Purity of compound lots used in bioassays was ≥90% unless otherwise noted (see Table S2).

#### 4.1.2. Synthesis of Dipeptides **9**–**12**

As noted in the text, procedures were adapted to meet the resources available in different laboratories. Methods used in some of the curricular laboratories are reported here, and variations on these are described in full in the Supporting Information. Purity and identity of all synthetic dipeptides were confirmed by LC-MS. Purity of compound lots used in bioassays was typically ≥90% unless otherwise noted. The following compounds have been previously reported: **9j** [15], **10b** [10,48,49], **10f** [50], **11b** [15], **11c** [51], **11d** [51,52], **11f** [51], **11h** [53], **11k** [54], **11p** [55,56], **11q** [57]. LC-MS and ^1^H NMR data for representative compounds **9a**, **9b**, **9c**, **9k**, **10b**, **11b**, **12b**, and **12c** are included in the Supporting Information.

Each vessel in a BillBoard solid-phase synthesis set (Chemglass, Vineland, NJ, USA) was loaded with 50 µmol of an Fmoc-protected amino acid on Wang resin (100–200 mesh, 1% DVB, Peptides International, Louisville, KY, USA) and washed with NMP (3 × 2 mL, Thermo Fisher, Waltham, MA, USA), allowing 30 s of gravity flow followed by gentle air pressure. Fmoc deprotection was achieved with successive washes with 20% piperidine (Thermo Fisher, Waltham, MA, USA) in NMP (3 × 2 mL) with gravity-only draining followed by 5 min incubations. After the final wash and incubation, remaining solvent was removed with gentle air pressure. NMP rinses (3 × 3 mL) were done with gentle air pressure. For acylation reactions the bottoms of each vessel were capped before loading with 0.6–1 mL of 0.25 M of a Boc-(S)-F-Phe (250 µmol, 3–5 equiv, Peptides International, Louisville, KY, USA) in 0.25 M HOBt (3–5 equiv, Sigma Aldrich, St. Louis, MO, USA) in NMP, followed by 0.3–0.5 mL 0.50 M DIC (3–5 equiv, Sigma Aldrich, St. Louis, MO, USA) in NMP. Reaction vessels were capped, mixed by inverting, and incubated for 2–7 days. Before cleavage reactions the reaction vessel bottom caps were carefully removed after inverting the vessels, subsequently turning them upright and agitating to effect solvent removal of resin adhering to the top caps before their removal. Acylated resin products were washed with NMP (3 × 2 mL), THF (3 × 2 mL, Thermo Fisher, Waltham, MA, USA), and finally with CH_2_Cl_2_ (4 × 2 mL, Thermo Fisher, Waltham, MA, USA), allowing only gravity filtration for each. With each vessel sitting on a tared (to 0.1 mg) and labeled vial, TFA (Sigma Aldrich, St. Louis, MO, USA)/CH_2_Cl_2_/H_2_O (2 mL, 35:60:5, *v*/*v*) were added, allowing 30 min for cleavage and gravity draining. Resins were rinsed with TFA/CH_2_Cl_2_/H_2_O (2 mL, 35:60:5, *v*/*v*) and CH_2_Cl_2_ (2 mL), draining completely with air pressure. Each filtrate was mixed and a 100 μL aliquot was removed and evaporated to dryness under N_2_ for LC/MS analysis. Remaining filtrate volumes were similarly evaporated to dryness and weighed. The crude products were purified using a 500 mg HyperSep Si cartridge (Thermo Fisher, Waltham, MA, USA) (iPrOH/MeOH/NH_4_OH, 18:2:1) to yield the targeted dipeptide product.

### 4.2. P. aeruginosa Bacterial Growth and Biofilm Formation Measurements

As noted in the text, slightly varied procedures were employed in mentored research laboratories and curricular laboratories. Methods used in mentored research are reported here; these were used to collect the data presented in Table 2. Variations on these are described in full in the Supporting Information, including the methods used to collect the biofilm formation measurements reported in Table 1, Tables S2 and S3.

Biofilms were formed by a modification of the multiwell plate assay [35] with *P. aeruginosa* strain PA14 (provided by Department of Biology, Indiana University Indianapolis) [58]. PA14 cultures were grown overnight in LB liquid medium (Sigma Aldrich, St. Louis, MO, USA) at 37 °C with shaking. The cultured PA14 was then diluted 1:1000 into M63 medium (15.1 mM (NH_4_)SO_4_, 40.2 mM K_2_HPO_4_, 22mM KH_2_PO_4_) (Sigma Aldrich, St. Louis, MO, USA), which had been completed with addition of 0.4% (*w*/*v*) arginine (MilliporeSigma, St. Louis, MO, USA) and 1 mM magnesium sulfate (complete M63 media). Experimental compounds (from stocks prepared in 10% *v/v* DMSO) were evaluated at various concentrations in duplicate alongside tobramycin (Sigma Aldrich, St. Louis, MO, USA) in 24-well plates. Wells with bacteria alone and media alone were also included. The plates were covered and incubated at 37 °C for 24 h, then absorbance at 600 nm was measured on a Tecan Infinite 200 PRO plate reader (Männedorf, Switzerland) to detect bacterial growth.

To perform the crystal violet staining for biofilms, the liquid in the wells was first aspirated. The wells were then washed twice with 1000 μL of water and then treated with 750 μL each of a 0.1% *v*/*v* crystal violet solution for 10 min. The stain solution was aspirated, and the wells were washed three times with 1000 μL of water, decanting each time. The plates were allowed to dry for 15 min, and the wells were then treated with 30% *v*/*v* acetic acid. After 15 min the dissolved biofilm liquid was scanned spectrophotometrically for absorbance at 600 nm. After subtracting the background absorbance, data were analyzed by normalizing individual samples to the average absorbance of untreated bacteria.

### 4.3. Human Cell Toxicity Assay

Cell lines Ramos, Jurkat, and THP-1 were all obtained from ATCC (Manassas, VA, USA) and maintained in a 37 °C, 5% CO_2_ humidified incubator. Cell lines were cultured in RPM1 1640 supplemented with 10% heat-inactivated fetal bovine serum and 1X penicillin-streptomycin (Corning, Corning, NY, USA). Cells (2 × 10^5^ cells/mL) were cultured in serial dilutions of test compounds, 25 µM etoposide, or equivalent amounts of vehicle (DMSO; 1:1000) [59]; each condition was run in quadruplicate. After 48 h, cell viability was measured using the Promega CellTiter 96 Aqueous One Solution Cell Proliferation Assay (Fitchburg, WI, USA), per the manufacturer’s directions, on a Tecan Infinite 200 Pro plate reader.

## 5. Conclusions

These studies demonstrate the promising potential of fluorinated unnatural dipeptides as novel antibiofilm compounds. A set of over 100 fluorinated unnatural dipeptides was synthesized and evaluated for inhibition of *P. aeruginosa* biofilm formation. Several SAR trends emerged, including the discovery that potent activity was associated with the natural (*S*)-configuration of fluorophenylalanine residues. Additionally, dipeptides containing 4-F-Phe or 3,4-diF-Phe combined with small hydrophobic amino acids exhibited the strongest antibiofilm activity. Further evaluation of the most potent 4-F-Phe dipeptide, **11b**, alongside its corresponding 3,4-diF-Phe analog, **12b**, revealed divergent profiles between the mono- and difluorinated analogs. Whereas **11b** inhibited both planktonic growth and biofilm formation, **12b** selectively inhibited biofilm formation without affecting bacterial growth, suggesting a distinct antivirulence mechanism. The similar biological profiles of these dipeptides and their corresponding fluorinated amino acids are consistent with a prodrug hypothesis, which will be further evaluated in future studies. Importantly, both compounds showed no significant cytotoxicity toward mammalian cell lines, highlighting their potential as selective leads for the development of new strategies to combat *P. aeruginosa* infections.

In parallel, the work presented here highlights the value of adaptable and scalable drug discovery projects that involve students through curricular laboratory modules. Our synthetic approaches have prioritized flexible but robust procedures, and these have been successfully applied by hundreds of students in different laboratory environments to generate a large diversity of compounds for study and evaluation. This approach simultaneously benefits the research outcomes and student learning. It is our hope that this work serves as a motivation for other educator-scholars to identify opportunities for students to contribute to research outcomes through their required laboratory coursework, and that our successes can motivate them to apply these practices at their own institutions.

## Figures and Tables

**Table 1:** *P. aeruginosa* antibiofilm screening of dipeptides **9**, **10**, **11**, and **12** at 1 μg/mL.

	N-Terminus								
	2-F-Phe		3-F-Phe		4-F-Phe		3,4-Di-F-Phe		
C- Terminus	Cmpd ID	Biofilm Formation ^a^ (%)	Cmpd ID	Biofilm Formation^a^ (%)	Cmpd ID	Biofilm Formation ^a^ (%)	Cmpd ID	Biofilm Formation ^a^ (%)	(%)
Gly	**9a**	55	**10a**	90	**11a**	22	**12a**	23	1
Ala	**9b**	79	**10b**	70	**11b**	14	**12b**	44	
Val	**9c**	29	**10c**	81	**11c**	37	**12c**	46	
Leu	**9d**	53	**10d**	75	**11d**	59	**12d**	27	
Ile	**9e**	61	**10e**	77	**11e**	56	**12e**	78	
Phe	**9f**	ND	**10f**	85	**11f**	66	**12f**	85	
Tyr	**9g**	86	**10g**	91	**11g**	89	**12g**	ND	
Ser	**9h**	56	**10h**	84	**11h**	70	**12h**	69	
Thr	**9i**	53	**10i**	87	**11i**	38	**12i**	61	
His	**9j**	98	**10j**	ND	**11j**	65	**12j**	ND	
Arg	**9k**	51	**10k**	100	**11k**	97	**12k**	86	
Lys	**9l**	ND	**10L**	76	**11L**	73	**12l**	90	
Met	**9m**	93	**10m**	100	**11m**	49	**12m**	72	
Asp	**9n**	ND	**10n**	105	**11n**	96	**12n**	ND	
Glu	**9o**	74	**10o**	88	**11o**	62	**12o**	55	
Gln	**9p**	ND	**10p**	105	**11p**	75	**12p**	ND	
Pro	**9q**	99	**10q**	94	**11q**	82	**12q**	100	100

^a^ Percent biofilm formation relative to control following Method A (Supporting Information); additional information, including compound lots and standard errors (SE) for biological replicates, is provided in Table S3 (Supporting Information).

**Table 2:** Inhibition of *P. aeruginosa* growth and biofilm formation and cytotoxicity in human Ramos, Jurkat, and THP-1 cells of compounds **3**, **4**, **11b**, and **12b**.

	IC_50_ (μg/mL) ^a^		IC_90_ (μg/mL) ^a^		CC_50_ (μg/mL) ^a,b^			
Compound	*P. aeruginosa* Growth	*P. aeruginosa* Biofilm Formation	*P. aeruginosa* Growth	*P. aeruginosa* Biofilm Formation	Ramos Viability	Jurkat Viability	THP-1 Viability	SI ^c^
(*S*)-4-F-Phe (**3**)	0.42	0.26	1.24	0.84	22.1	>100	>100	53
(*S*,*S*)-4-F-Phe-Ala (**11b**)	0.57	0.36	3.46	2.95	>100	>100	>100	>175
(*S*)-3,4-diF-Phe (**4**)	>100	0.08	>100	1.81	>100	>100	>100	–
(*S*,*S*)-3,4-diF-Phe-Ala (**12b**)	>100	0.17	>100	2.43	>100	>100	>100	–

^a^ IC_50_, IC_90_, and CC_50_ values were calculated using the following resource: “Quest Graph™ IC50 Calculator.” AAT Bioquest, Inc., (Pleasanton, CA, USA) https://www.aatbio.com/tools/ic50-calculator (accessed on 12 February 2026). ^b^ CC_50_: Cytotoxic Concentration that reduces cell viability by 50% in a 48 h assay; ^c^ SI: Selectivity Index, calculated as Ramos CC_50_/*P. aeruginosa* growth IC_50_; values for **4** and **12b** were not included because bacterial growth inhibition IC_50_ could not be measured.

## Data Availability

Data are contained within the article and Supporting Information. Further inquiries can be directed to the corresponding authors.

## Supplementary Materials

The following supporting information can be downloaded at: https://www.mdpi.com/article/10.3390/molecules31132341/s1, Table S1. Locations, laboratory environments, and methods used for synthesis and biological screening of dipeptides; Figure S1. The Bill-Board synthesis apparatus; Scheme S1. Synthesis of dipeptides **5**–**12** (same as Scheme 1 in manuscript); Scheme S2. Synthesis of dipeptides with (*RS*)-fluorophenylalanine residues; Scheme S3. Loading fluorophenylalanine amino acids onto Wang resin for synthesis of dipeptides; Figure S2. Comparison of biofilm formation of *P. aeruginosa* treated with 20 µg/mL racemic non-natural amino acid analogs (Method A); Table S2. Representative dipeptide synthesis and *P. aeruginosa* biofilm formation measurements for **5**–**8**; Table S3. Summary of dipeptide synthesis and *P. aeruginosa* biofilm formation measurements for **9**–**12**; Additional details on methods used for synthesis and biological evaluations of dipeptides; characterization data of representative dipeptides. References [15,21,33,35,58] are also cited in the Supplementary Materials.

## Abbreviations

The following abbreviations are used in this manuscript:

Boc	*tert*-butoxycarbonyl
BTPP	*tert*-butylimino-tri(pyrrolidino)phosphorane
CC_50_	Cytotoxic concentration (reduces cell viability by 50%)
CURE	Course-based undergraduate research experience
D3	Distributed Drug Discovery
DCM	dichloromethane
DIC	*N*,*N*′-diisopropylcarbodiimide
DIPEA	*N*,*N*-diisopropylethylamine
DMF	*N*,*N*-dimethylformamide
DMSO	dimethylsulfoxide
DVB	divinylbenzene
Fmoc	9-fluorenylmethoxycarbonyl
HOBt	1-hydroxybenzotriazole
IC_50_	Inhibitory concentration (reduces biological activity by 50%)
LB	Lysogeny broth
LC	Liquid chromatography
MBC	Minimum bactericidal concentrations
MBEC	Minimum biofilm eradication concentration
MBIC	Minimum biofilm inhibitory concentration
Me	methyl
MIC	Minimum inhibitory concentration
MS	Mass spectrometry
NMP	*N*-methyl-2-pyrrolidone
Pr	propyl
SEM	Scanning electron microscope
SI	Selectivity index
T3P	propanephosphonic acid anhydride
TFA	trifluoroacetic acid
THF	tetrahydrofuran
