# Supplementary material for: Fluorophenylalanine-Containing Dipeptides with Antibiofilm Activity Against Pseudomonas aeruginosa Discovered Through a Collaborative Undergraduate Research and Educational Program
Source: Molecules. 2026 Jul 3;31(13):2341. doi: 10.3390/molecules31132341 (PMC13363317; doi:10.3390/molecules31132341)
Supplement: Supplementary file 1 [file molecules-31-02341-s001.zip › molecules-4389786-supplementary.pdf]

Supporting Information

Fluorophenylalanine-Containing Dipeptides with Antibacterial Activity Against *Pseudomonas aeruginosa* Discovered through a Collaborative Undergraduate Research and Educational Program

Amy B. Dounay,[1†*] Amelia A. Fuller,[2†*] Dylan R. Y. Lawton,[2] Sarah Buchman,[3] Markus F. Bergstrom,[1] Rose Gilmore,[4] Emerson Hernly,[5] Jake Hitchens,[5] Justin Tee,[1] Callista Tran,[1] Nathan Weiler,[4] Gregory G. Anderson,[4] Olivia Hatton,[3] Kathleen A. Marrs,[4] Kristen Mudrack,[6] Martin J. O'Donnell,[5‡] Doug Schirch,[7] Kevin Sullivan,[8] J. Geno Samaritoni,[5] William L. Scott.[5]

1 Department of Chemistry and Biochemistry, Colorado College, 14 E. Cache La Poudre Street, Colorado Springs, CO 80903 USA

2 Department of Chemistry and Biochemistry, Santa Clara University, 500 El Camino Real, Santa Clara, CA 95053 USA

3 Department of Molecular Biology, Colorado College, 14 E. Cache La Poudre Street, Colorado Springs, CO 80903 USA

4 Department of Biology, Indiana University Indianapolis, 723 W. Michigan Street, SL 330, Indianapolis, IN 46202, USA

5 Department of Chemistry and Chemical Biology, Indiana University Indianapolis, 402 N. Blackford St., LD 326, Indianapolis, IN 46202, USA

6 Department of Chemistry, Milligan University, 1 Blowers Boulevard, Milligan, TN 37682, USA

7 Department of Chemistry, Goshen College, 1700 South Main Street, Goshen, IN 46526, USA

8 Department of Chemistry, University of Indianapolis, 1400 East Hanna Avenue, Indianapolis, IN 46227 USA

† Both authors contributed equally.

‡ Deceased August 1, 2023.

* Correspondence: aafuller@scu.edu, adounay@coloradocollege.edu

Table of Contents

**Table S1.** Locations, laboratory environments, and methods used for synthesis and biological screening of dipeptides	3
General methods and abbreviations	4
BillBoard synthesis apparatus and example synthesis array<br>**Figure S1.** The BillBoard synthesis apparatus. A) picture of the BillBoard equipment. B) example synthesis array of dipeptides.	5
Synthesis Methods<br>**Scheme S1.** Synthesis of dipeptides **5-12** (same as Scheme 1 in manuscript).<br><br>Synthesis of Dipeptides **5-8**<br>*Method X*<br>**Scheme S2.** Synthesis of dipeptides with (*R/S*)-fluorophenylalanine residues.<br>*Method Y*<br>**Scheme S3.** Loading fluorophenylalanine amino acids onto Wang resin for synthesis of dipeptides.<br>*Method Z*<br><br>Synthesis of Dipeptides **9-12**<br>*Method A*<br>*Method B*<br>*Method C*	6-11<br><br><br>6-9<br>6<br>6<br><br>7<br>7<br><br>8<br><br>9-10<br>9<br>9<br>10
LCMS analysis of crude dipeptides	11
Purification and identification of dipeptides **9-12**	12
Characterization data of selected compounds prepared	12-21
Biological evaluations methods	22
**Figure S2.** Comparison of biofilm growth of *P. aeruginosa* treated with 20 μg/mL racemic non-natural amino acid analogs.	23
**Table S2.** Representative dipeptide synthesis and *P. aeruginosa* biofilm inhibition studies for **5-8**.	24-26
**Table S3.** Summary of dipeptide synthesis and *P. aeruginosa* biofilm growth inhibition for **9-12**.	27-29
References	30

Table S1. Locations, laboratory environments, and methods used for synthesis and biological screening of dipeptides

Institution	Chemistry laboratory environment(s)	Dipeptide scaffolds prepared	Synthesis and purification method(s) used	Biology laboratory environment
Indiana University, Indianapolis	Undergraduate laboratory course and mentored research	**5-12**	**C, 2**	Undergraduate laboratory course and mentored research
Santa Clara University	Mentored research	**5-12**	**A, Z, 1**	N/A
Colorado College	Undergraduate laboratory course and mentored research	**9-12**	**B, 2**	Mentored research
Goshen College	Mentored research	**6, 7, 12**	**C, 2**	N/A
University of Indianapolis	Laboratory course	**10, 11**	**C, 2**	N/A
Milligan University	Mentored research	**5-7**	**C, 2**	Mentored research
University of Puerto Rico at Río Piedras	Laboratory course	**10, 11**	**C, 2**	N/A
Universidad Nacional Autónoma de México	Laboratory course	**10, 11**	**C, 2**	N/A

General methods and abbreviations

Chemicals and solvents described were ordered from common suppliers, including Aldrich, Aapptec, Peptides International, etc., and used without further purification. We note that different institutions and laboratory environments used materials from different suppliers.

Abbreviations used in the procedures are:

**Abbreviation/ Acronym**	**Chemical Name**
Boc	*tert*-butoxycarbonyl
BTPP	*tert*-butylimino-tri(pyrrolidino)phosphorane
DCM	dichloromethane
DIC	*N,N'*-diisopropylcarbodiimide
DIEA	*N,N*-diisopropylethylamine
DMF	*N,N*-dimethylformamide
DVB	divinylbenzene
Fmoc	9-fluorenylmethoxycarbonyl
HOBt	1-hydroxybenzotriazole
Me	methyl
NMP	*N*-methyl-2-pyrrolidinone
Pr	propyl
T3P	propanephosphonic acid anhydride
TFA	trifluoroacetic acid
THF	tetrahydrofuran

BillBoard synthesis apparatus and example synthesis array

BillBoards are spatially addressable 3 x 2 arrays used for parallel synthesis of dipeptides that were employed by some of the laboratories engaged in this work (Figure S1). The BillBoard includes six solid-phase reaction vessels and a drain tray to collect excess reagents and solvent washes. BillBoards are commercially available through ChemGlass (https://chemglass.com/bill-board-solid-phase-syntheses-sets). An example synthesis array is shown in Figure S1 (panel B).

Figure S1. The BillBoard synthesis apparatus. A) Picture of the BillBoard equipment. B) Example synthesis array of dipeptides.

A)

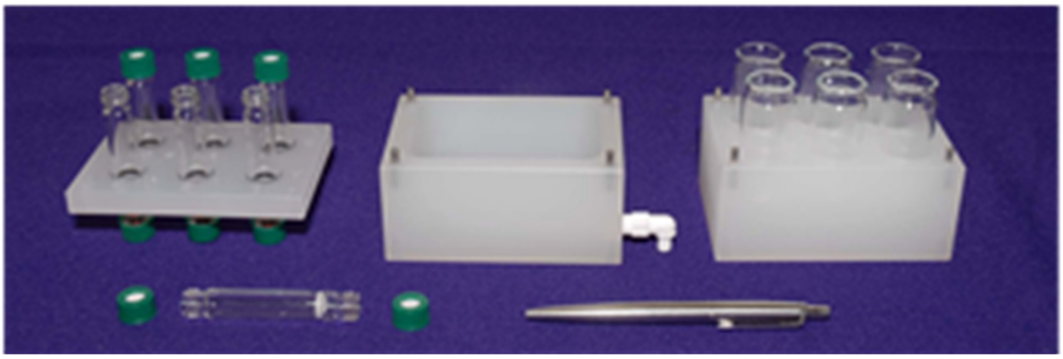

BillBoard and Reaction Vessel **BillBoard Drain Tray** **Vial Collection Rack**

B)

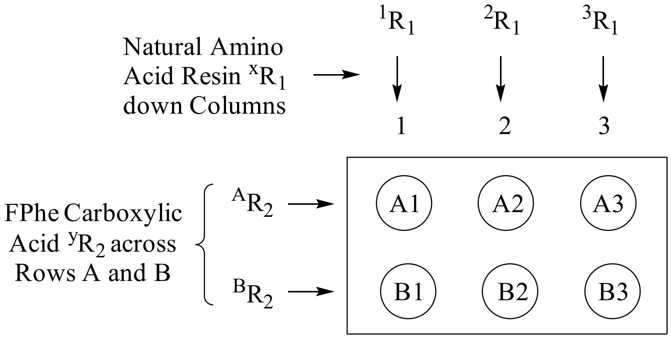

Synthesis Methods

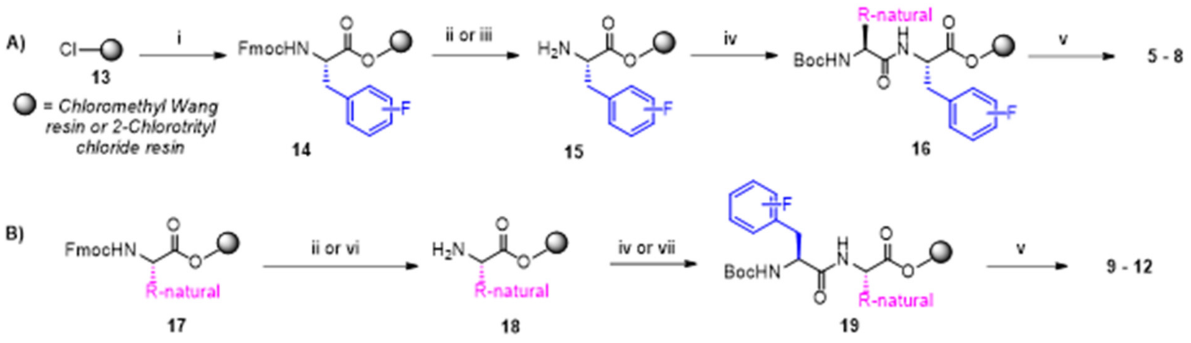

Scheme S1. Synthesis of dipeptides **5-12** (same as Scheme 1 in manuscript).

Synthesis of dipeptides **5-8**

Method X

Scheme S2. Synthesis of dipeptides with (*R/S*)-fluorophenylalanine residues

Fmoc-Gly-Wang resin (8.71 g, 6.10 mmol) was swelled for 30 min with 70 ML of NMP in a 250-mL solid-phase peptide synthesis vessel under dry argon gas. The vessel was drained and the resin was treated with 35 mL of 20% piperidine in NMP for 2 min. The vessel was drained and the resin was treated with 85 mL of 20% piperidine and rocked for 45 min on an orbital shaker. The vessel was drained, and the resin was washed with 5 x 70 mL x 2 min NMP. The deprotected resin was then treated with 10.24 mL of benzophenone imine in 50 mL of NMP, followed by 3.04 mL of acetic acid in 50 mL of NMP. The vessel was rocked overnight at room temperature. After 21 h, the vessel was drained and the resin was washed with 3 x 65 mL x 2 min NMP and 4 x 65 x 2 min dichloromethane each. Resin **1** was dried under a slow stream of dry nitrogen gas for approximately 30 h and then was stored at 2 °C.

50 mmols of resin **SI-1** was treated with 100 mmols of 0.20 M BTPP in NMP, followed by 100 mmols of fluorinated benzyl bromide in NMP. After 7 days, the reaction mixture was filtered and the resulting resin **SI-2** was washed once with 3 mL of THF. To the resin was then added 2.5 mL of 1.0 N hydrochloric acid in THF (1:2). After 20 min, the resin was filtered and washed with 3 mL of THF followed by 2 x 2.5 mL x 5 min of 0.20 M diisopropylethylamine in NMP, and 2 x 2/5 mL of NMP to give resin **SI-3**. Resins 3 were treated with 250 mmols each of a Boc-protected amino acid and HOBt (0.25 M each in NMP), followed by 250 mmols of 0.50 M DIC in

NMP. After standing 2-5 days, the resins were filtered and washed with 3 x 2 mL each of NMP and THF and 3 x 2 mL each of dichloromethane to give resins **SI-4**. Treatment of resins 4 with 2 mL of 35:60:5 TFA/DCM/H_2O (trifluoroacetic acid/dichloromethane/water) for 30 minutes (drip cleavage) was followed by washing the resin with 2 mL of 35:60:5 TFA/DCM/H_2O and 2 mL of DCM. The combined filtrates were evaporated to give crude salts **SI-5**.

Method Y

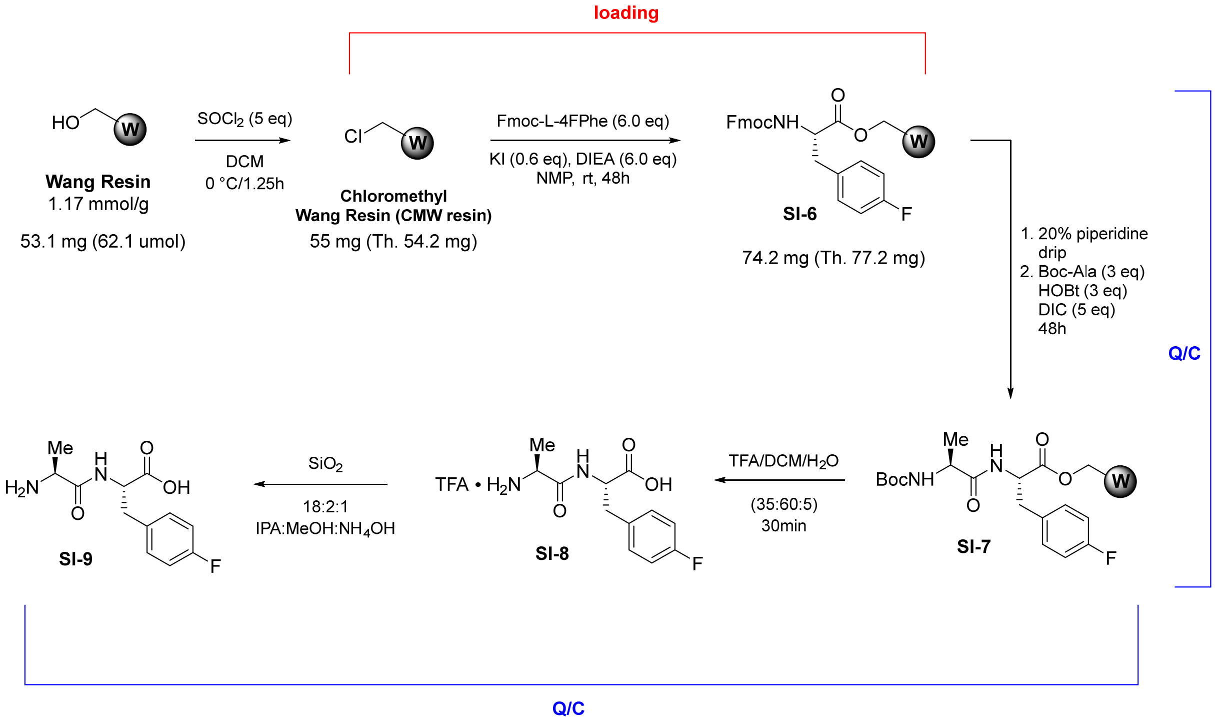

Scheme S3. Loading fluorophenylalanine amino acids onto Wang resin for synthesis of dipeptides.

Loading of Fmoc-(S)-4-fluorophenylalanine onto Wang resin. A 25-mL, 2-neck, round-bottom flask with stir bar and fitted with a gas inlet tube was assembled while hot and was purged with a slow stream of dry nitrogen gas. The flask was then charged with 53.1 mg (62.1 μmol) of Wang resin (Advanced ChemTech SA5009) and 4 mL of dichloromethane (DCM). The contents were cooled with an ice bath and then 22.5 μL (36.9 mg, 310 μ mol, 5.00 equiv.) of thionyl chloride was added in one portion while stirring. After 1.25 h at 0 °C with occasional stirring, the contents were transferred using DCM and a disposable pipet to a 5.0-mL SPPS vessel. The resin was then washed with 8 × 2 mL of DCM followed by 8 × 2 mL of methanol. The resin was briefly dried using a stream of dry nitrogen gas and then under house vacuum at room temperature for two hours to give 55 mg of the chloromethyl Wang resin (CMW resin).

To the CMW resin contained in the SPPS vessel was added 149.8 mg (369 μmol, 6.0 equiv) of Fmoc-*(S)*-4-fluorophenylalanine in 750 uL of NMP, 6.4 mg (38.5 μmol, 0.62 equiv.) of potassium iodide in 0.25 mL of NMP, and 64.4 μL (47.8 mg, 370 μmol, 6.0 equiv) of diisopropylethylamine in 0.50 mL of NMP. The vessel was gently agitated for 48h on a rotator assembly. The vessel

was then drained and the resin and inside wall of the vessel were washed with 0.50 mL of NMP. The combined filtrates were set aside for recovery of unreacted Fmoc-*(S)*-4-fluorophenylalanine. The resin was then washed with 3 mL each of NMP and methanol followed by 2 × 3 mL of NMP, 2 × 6 mL of water, 3 mL of methanol, 2 × 3 mL of DCM, and 2 × 3 mL of methanol. The resin was then dried under a stream of dry nitrogen gas and then under house vacuum overnight to afford 74.2 mg (Th. 77.2 mg) of resin **SI-6**.

Q/C of Resin 1. Resin **SI-6** was washed with 2 × 2 mL of NMP and was then swelled in 2 mL of NMP for 25 min. The vessel was drained and the resin was washed with 2 × 2 mL of NMP. The Fmoc resin **SI-6** was then treated with 4 × 2 mL × 5 min of 20% (v/v) piperidine in NMP. The deprotected resin was then washed with 8 × 2 mL of NMP. To the resin was then added a solution of 35.8 mg (187 µmol, 3.00 equiv.) of 1-hydroxybenzotriazole monohydrate and 35.3 mg (187 µmol, 3.00 equiv.) of Boc-Ala in 1.5 mL of NMP followed by 28.8 µL (23.5 mg, 186 µmol, 5.0 equiv.) of *N,N'*-diisopropylcarbodiimide in 0.5 mL of NMP. The vessel was then rotated overnight. After 20 h the vessel was drained and the resin was washed with 3 × 2 mL each of NMP and THF and 6 × 2 mL of DCM to give resin **SI-7**.

2 mL of 35/60/5 (v/v/v) trifluoroacetic acid/dichloromethane/water was added to resin **SI-7** **c**ontained in the 5 mL SPPS vessel, and the vessel was rotated for 30 min. The vessel was drained into a weighed collection vial, and the resin was washed with 1 × 2 mL each of the cleavage cocktail and DCM. The solution was evaporated to give 24.2 mg (Th. 22.9 mg) of the TFA salt **SI-8**; ^{1}HNMR (D_2O) d 1.46 (d, 3H, *J* = 7.1 Hz), 3.05 (dd, 1H, *J* = 14.2 and 8.9 Hz), 3.22 (dd, 1H, *J* = 14.1 and 5.7 Hz), 3.99 (q, 1H, *J* = 7.2 Hz), 4.64 (dd, 1H, *J* = 8.8 and 5.8 Hz), 7.09 (t, 2H, *J* = 8.9 Hz), 7.26 (dd, 2H, *J* = 8.5 and 5.6 Hz). This sample was dissolved in 250 uL of 18/2/1 IPA/MeOH/NH_4OH and was applied to a 500-mg cartridge of HyperSep SI silica gel, which had been equilibrated with 18/2/1 IPA/MeOH/NH_4OH. The column was eluted with 18/2/1 IPA/MeOH/NH_4OH to afford 10.0 mg (63% overall from Wang resin) of free base **SI-9**. LCMS rt 1.63 min, 98% purity at 210 nm; ESI-MS, *m/z* 255 [M+H].

Method Z

Variants of **7** and **8** were also synthesized following solid-phase synthesis techniques. Chlorotrityl chloride resin (0.1 mmol) was added to a 20 mL synthesis vessel equipped with a coarse ground glass frit and suspended in 3 mL DCM for 30 minutes. The DCM was drained from the resin. A solution of Fmoc-4-F-Phe-OH (for the synthesis of **7**) or Fmoc-3,4-diF-Phe-OH (for synthesis of **8**) (0.1 mmol) in DIEA (0.4 mmol, 0.070 mL, 4 equiv) in DCM (10 mL DCM per gram of protected amino acid solute) was mixed by vortex until dissolved, then added to the swelled resin. The reaction vessel was capped and shaken on a wrist-action shaker for 60 minutes at room temperature. The solution was drained and discarded, and the resin was rinsed three times with DCM. A 3 mL solution of DCM, methanol, and DIEA (80:15:5, 18 mL total) was added to the reaction vessel. The reaction vessel was then capped and shaken for 20 minutes at room temperature. The solution was drained and discarded and rinsed three times each with DMF and DCM. A solution of 30% 4-methylpiperidine in DMF (3 mL) was added to the reaction vessel, and the vessel was capped and shaken at room temperature for 10 minutes then drained. The addition of the 4-methylpiperidine solution, shaking and draining were repeated two more times. A small portion of the filtrate was saved after each round of shaking and spotted onto a silica TLC plate with a capillary. Visualization of a UV-active spot using a UV lamp allowed identification of fluorene, the product of Fmoc-removal and thus confirmed successful attachment of the amino acid to the resin.

The resin was washed three times with DMF and drained. A solution of *N*-Fmoc-protected amino acid residue (0.4 mmol, 4 equiv), HBTU (0.36 mmol, 3.6 equiv), DIEA (0.8 mmol, 8 equiv), in 1.48 mL DMF was mixed for 5 minutes, then added to the reaction vessel. The reaction mixture was capped and shaken for 2 hours. The filtrate was drained and discarded, and the resin was washed three times with DMF. The resin was then treated with the same deprotection step using 30% 4-methylpiperidine in DMF and testing of the filtrate again confirmed the addition of the Fmoc-protected amino acid. The resin was washed sequentially three times each with DMF, THF, MeOH, DCM, MeOH, and DCM. To cleave the dipeptide from the resin, a 5 mL solution of TFA, DCM, and H_2O (35:60:5) was added to the resin, agitated gently, and allowed to equilibrate for one hour. The filtrate containing the dipeptide was collected into a flask, and the resin was rinsed with 2 mL of the cleavage solution and 2 mL DCM. The combined filtrates were transferred to a 20 mL scintillation vial for further analyses. A small amount of the filtrate was saved in a 20 μL LC-MS vial for LC-MS analysis, and the remainder of the filtrate was then placed on a rotavapor and lyophilizer and stored until HPLC analysis.

Synthesis of Dipeptides **9-12**

Method A

Variants of **9 - 12** were synthesized following a standard solid-phase peptide synthesis technique modified from Dounay et al.[1] Wang resins pre-loaded with *N*-Fmoc-protected amino acids (0.1 mmol) were added to a 20 mL synthesis vessel equipped with a coarse ground glass frit and suspended in DMF (3 mL) for 30 minutes. The DMF was drained and a 20% 4-methylpiperidine in DMF solution (3 mL) was added to the swelled resin. The capped reaction vessel was shaken for 15 minutes on a wrist-action shaker at room temperature. The solution was drained, and this deprotection step was repeated once more. The solution was drained from the reaction vessel, and the resin was rinsed with DMF and NMP (5 × each). To the deprotected resin was added three solutions: 0.25 M Boc-3-F-Phe-OH (5 equiv) in NMP (2 mL), 0.25 M HOBt (5 equiv) in NMP (2 mL), and 0.5 M DIC (5 equiv) in NMP (1 mL). The capped reaction vessel was shaken for 30 minutes. The solution in the chamber was drained, and the resin was rinsed five times with NMP (5 volumes), then with THF and DCM (3 × each). To cleave the dipeptide from the Wang resin and remove any side chain protecting groups on the *C*-terminal residue, a solution of TFA, DCM, and H_2O (35:60:5, 5 mL) was added to the resin, agitated gently, and allowed to equilibrate for one hour. The filtrate containing the dipeptide was collected into a flask, and the resin was rinsed with an additional portion of the cleavage solution, then DCM (2 mL each). The combined rinses were added to the filtrate and transferred to a 20 mL scintillation vial for further analyses.

Method B

These methods were reported by Tran et al.[2] Each reaction vessel in a BillBoard solid phase synthesis apparatus was capped at the bottom and loaded with Fmoc-amino acid-Wang resin (0.050 mmol). Ethyl acetate (~1.5 mL) was added, and the mixture was allowed to sit for 10 – 15 min. The vessels were uncapped at the bottom, and the solvent was allowed to drain. A freshly prepared solution of 20% piperidine in ethyl acetate (v/v, 2 mL) was added and the mixture was allowed to sit for 5 min while the solution drained by gravity. This step was repeated two additional times with fresh portions of 20% piperidine in ethyl acetate (2 mL). The resin was washed with ethyl acetate (5 x 2 mL).

The bottoms of the reaction vessels were recapped. To each reaction vessel, 1 mL of the appropriate Boc-F-Phe solution was added (0.25 mmol Boc-F-Phe, 5 equiv; 0.25 mmol HOBt, 5

equiv) followed by 0.2 mL of commercially prepared T3P in ethyl acetate (50% w/v, 1.7 M, 3 mmol, 6 equiv). The tops of the reaction vessels were capped, and the vessels were inverted a few times to effect mixing. The BillBoard was allowed to sit at room temperature for 18 - 72 h. The reaction vessels were uncapped at the top and bottom, and the resin was washed with ethyl acetate (5 x 2 mL) and CH_2Cl_2 (3 x 2 mL).

The BillBoard was placed over collection vials, and each vessel was treated with a solution of TFA/ CH_2Cl_2/H_2O (35:60:5, 2 mL). The BillBoard was allowed to sit without agitation for 30 min and the eluate was drained by gravity into the collection vials. Each reaction vessel was rinsed with an additional cleavage solution (2 mL), then CH_2Cl_2 (2 mL), and the eluate was collected into the collection vials. A small aliquot of the eluate (100 mL) was evaporated to dryness and analyzed by LC/MS. The remainder was concentrated by evaporation under a stream of N_2 (effluent TFA vapor was scrubbed in a caustic solution of sodium hydroxide). The crude products were purified using a 500 mg HyperSep Si cartridge (iPrOH/MeOH/NH_4OH, 18:2:1) to yield the targeted dipeptide product. A more non-polar starting ratio was sometimes needed for non-polar dipeptides.

Method C

Each vessel in a BillBoard solid-phase synthesis set was loaded with 50 µmol of an Fmoc-protected amino acid on Wang resin (100-200 mesh, 1% DVB, Peptides International) and washed with 3 x 2mL NMP allowing 30 sec of gravity flow followed by gentle air pressure. Fmoc deprotection was achieved with successive 3 x 2 mL washes with 20% piperidine in NMP with gravity-only draining followed by 5 min. incubations. After the final wash and incubation, remaining solvent was removed with gentle air pressure. NMP rinses, 3 x 3 mL, were done with gentle air pressure. For acylation reactions the bottoms of each vessel were capped before loading with 0.6-1 mL of 0.25 M of a Boc-*(S)*-F-Phe (250 mmol, 3-5 equiv) in 0.25 M HOBt (3-5 equiv) in NMP, followed by 0.3-0.5 mL 0.50 M DIC (3-5 equiv) in NMP. Reaction vessels were capped, mixed by inverting, and incubated for 2-7 days. Before cleavage reactions the reaction vessel bottom caps were carefully removed after inverting the vessels, subsequently turning them upright and agitating to effect solvent removal of resin adhering to the top caps before their removal. Acylated resin products were washed with 3 x 2 mL NMP, 3 x 2 mL THF, and finally with 4 x 2 mL CH_2Cl_2, allowing only gravity filtration for each. With each vessel sitting on a tared (to 0.1 mg) and labeled vial, 2 mL TFA/CH_2Cl_2/H_2O (35:60:5, v/v) were added, allowing 30 min for cleavage and gravity draining. Resins were rinsed with 2 mL TFA/CH_2Cl_2/H_2O (35:60:5, v/v) and 2 mL CH_2Cl_2, draining completely with air pressure. Each filtrate was mixed and a 100 mL aliquot was removed and evaporated to dryness under N_2 for LC/MS analysis. Remaining filtrate volumes were similarly evaporated to dryness and weighed.

LCMS analysis of crude dipeptides

IUI methods:

Method 1: Performed using an Agilent Technologies 1200 Series HPLC fitted with an Eclipse XDB - C18 5 - micron column, 4.6x150 mm length, 5-microliter injections at a flow rate of 1.0 mL/min. A linear gradient from 20 % 1 :1 MeCN:MeOH (5 mM NH_4OAc) and 80 % water (5 mM NH_4OAc) to 100 % 1:1 MeCN:MeOH (5 mM NH_4OAc) over 10 minutes was used. Diode array detection (DAD) was performed at 210, 214, and 254 nm. Mass spectral analysis was performed on an Agilent Technologies 6130 Quadrupole LCMS using the electrospray-atmospheric pressure ionization method (ES-API) in the positive mode

Method 2: Performed using a Kinetex 2.6u XB – C18 50x2.1 mm column at 50 ° C, 1.0 mL/min, A: 0.1 % formic acid in water; B: 0.1 % formic acid in acetonitrile; 0.2 min at 5% B, 5-100 % B in 3.0 min; Hold 0.5 min at 100 % B.

Method 3: Performed using a 3.5m Waters X - Bridge C18 2.1x50 mm column at 50 ° C, 1.0 mL/min, A: 10 mM ammonium bicarbonate pH 10; B: acetonitrile; 0.2 min hold at 5 % B, 5-100 % B in 3.0 min, hold 0.5 min at 100 % B.

Colorado College method:

LC-MS analysis was performed using a Waters Acquity UPLC-MS system equipped with a BEH C18 2.1 x 50 mm, 1.7 µm packed column at 40.0 ˚C, QDa MS scan either 150 – 400 or 200 - 400 Da, and a photodiode array detector. Purity analysis was determined based on UV detection at 214 nm. Mobile phase A: 5 mM ammonium acetate in water; Mobile phase B: 5 mM ammonium acetate in 50% acetonitrile/50% methanol. Gradient 5% to 95% B linear over 3 min, and a hold at 95% B for 2 min; Flow rate of 0.6 mL/min. Injection volume of 1.00 µL.

Purification and Identification of dipeptides **9-12**

Method 1

Dipeptides were purified by reverse-phase high-performance liquid chromatography (RP-HPLC) on a Hitachi Chromaster 5000 instrument. A semi-preparative AAPPTec Spirit C18 column (5 mM, 10.0 mm x 25 cm) was used, and a linear gradient of solvents (A: 0.1% aqueous TFA; B: methanol) from 5-90% methanol, 40 minutes at a 3 mL/min flow rate was applied to separate and elute the dipeptides. Dipeptides were detected by UV absorbance at 220 nm and visualized with EZChrom software. Eluted compounds were collected in fractions, which were analyzed by electrospray ionization mass spectrometry on an Agilent QTOF6520 instrument. Those that contained the desired products were pooled, concentrated by rotary evaporation to remove the methanol, and then lyophilized to white powders.

Method 2

The crude products were purified using a 500 mg HyperSep Si cartridge (iPrOH/MeOH/NH_4OH, 18:2:1) to yield the targeted dipeptide product.

Characterization data of selected compounds prepared

General Methods

^{1}H NMR spectra were recorded on a Bruker AVANCE NEO Nanobay 400 MHz spectrometer with autosampler, and raw data were processed and analyzed using MestReNova (Mnova) software; chemical shifts are reported in parts per million (d ppm).

((*S*)-(2-amino-3-(2-fluorophenyl)propanoyl)glycine (9a). Prepared using Method A: ^{1}H NMR (400 MHz, D_2O) δ 7.32-7.38 (m, 1H), 7.27 (dt, 1H, *J* = 9.2, 7.7, and 1.7 Hz), 7.11-7.18 (m, 2H), 4.24 (t, 1H, *J* = 7.2 Hz), 3.88 (d, 1H, *J* = 17.5 Hz), 3.64 (d, 1H, *J* = 17.5 Hz), 3.25 (dd, 1H, *J* = 14.4 and 7.6 Hz), 3.20 (dd, 1H, *J* = 14.3 and 7.0 Hz); LCMS (crude) rt 0.39 min, 88% purity at 200-300 nm; ESI-MS *m/z* 241 [M+H], 239 [M-H].

((*S*)-2-amino-3-(2-fluorophenyl)propanoyl)-L-alanine (9b). Prepared using Method B: ^{1}H NMR (400 MHz, , D_2O) δ ^{1}H NMR (400 MHz, d_2o) δ 7.41 – 7.26 (m, 2H), 7.22 – 7.10 (m, 2H), 4.24 (t, *J* = 7.1 Hz, 1H), 4.09 (dd, *J* = 9.3, 6.3 Hz, 1H), 3.32 (dd, *J* = 14.4, 6.7 Hz, 1H), 3.19 (dd, *J* = 14.3, 7.5 Hz, 1H), 1.30 (d, *J* = 7.2 Hz, 3H), 1.22 (d, *J* = 9.2 Hz, 1H); LCMS rt 0.70 min, 100% purity at 214 nm; ESI-MS, *m/z* 252.7 [M-H].

((*S*)-2-amino-3-(2-fluorophenyl)propanoyl)-L-valine (9c). Prepared using Method B: ^{1}H NMR (400 MHz, D_2O) δ 7.42 – 7.32 (m, 1H), 7.32 – 7.24 (m, 1H), 7.21 – 7.11 (m, 2H), 4.34 (t, *J* = 6.7 Hz, 1H), 4.01 (d, *J* = 6.3 Hz, 1H), 3.32 (dd, *J* = 14.4, 6.6 Hz, 1H), 3.23 (dd, *J* = 14.4, 6.8 Hz, 1H), 2.01 (q, *J* = 6.7 Hz, 1H), 1.25 (d, *J* = 9.4 Hz, 2H), 1.19 (d, *J* = 6.3 Hz, 1H), 0.89 (dd, *J* = 8.9, 6.8 Hz, 6H); LCMS rt 1.01 min, 100% purity at 214 nm; ESI-MS, *m/z* 280.7 [M-H].

((*S*)-2-amino-3-(2-fluorophenyl)propanoyl)-L-arginine (9k). Prepared using Method A: ^{1}H NMR (400 MHz, D_2O) δ 7.22-7.33 (m, 2H), 7.08-7.16 (m, 2H), 4.10 (dd, 1H, *J* = 7.6 and 5.5 Hz), 3.76 (t, 1H, *J* = 6.4 Hz), 3.12 (t, 2H, *J* = 7.0 Hz), 3.02 (d, 2H, *J* = 6.4 Hz), 1.70-1.79 (m, 1H), 1.59-1.68 (m, 1H), 1.42 (quintet, 2H, *J* = 7.6 Hz); ^{13}C NMR (400 MHz, D_2O) δ 178.0, 175.3, 161.2 (d, ^1J = 244 Hz), 156.7, 131.9 (d, ^3J = 4.8 Hz), 129.1 (d, ^3J = 8.0 Hz), 124.4 (d, ^4J = 3.5 Hz), 123.6 (d, ^2J = 15.8 Hz), 115.3 (d, ^2J = 22.2 Hz), 55.3, 54.5, 40.7, 33.5, 29.0, 24.3; LCMS rt 3.38 min, 100% purity at 210 nm; ESI-MS, *m/z* 338.2 [M+H].

((*S*)-2-amino-3-(3-fluorophenyl)propanoyl)-L-alanine (10b). Prepared using Method A: ^{1}H NMR (400 MHz, D_2O) δ 7.35-7.40 (m, 1H), 7.05-7.10 (m, 3H), 4.22 (dd, 1H, *J* = 7.8 and 6.5 Hz), 4.08 (q, 1H, *J* = 7.2 Hz), 3.27 (dd, 1H, *J* = 14.3 and 6.4 Hz), 3.14 (dd, 1H, *J* = 14.3 and 7.9 Hz), 1.30 (d, 3H, *J* = 7.2 Hz); LCMS (crude) rt 0.53 min, 100% purity at 200-300 nm; ESI-MS *m/z* 255 [M+H], 253 [M-H].

((*S*)-2-amino-3-(4-fluorophenyl)propanoyl)-L-alanine (11b). Prepared using Method A: ^{1}H NMR (400 MHz, D_2O) δ 7.27-7.31 (m, 2H), 7.09-7.13 (m, 2H), 4.20 (dd, 1H, *J* = 7.5 and 6.5 Hz), 4.09 (q, 1H, *J* = 7.2 Hz), 3.25 (dd, 1H, *J* = 14.4 and 6.4 Hz), 3.14 (dd, 1H, *J* = 14.4 and 7.6 Hz), 1.31 (d, 3H, *J* = 7.2 Hz); LCMS (crude) rt 0.54 min, 100% purity at 200-300 nm; ESI-MS *m/z* 255 [M+H], 253 [M-H].

((*S*)-2-amino-3-(3,4-difluorophenyl)propanoyl)-L-alanine (12b). Prepared using Method B: ^{1}H NMR (400 MHz, D_2O) δ 7.33 – 7.20 (m, 2H), 7.14 – 7.06 (m, 1H), 4.22 (dd, *J* = 7.5, 6.5 Hz, 1H), 4.12 (q, *J* = 7.2 Hz, 1H), 3.27 (dd, *J* = 14.4, 6.5 Hz, 1H), 3.16 (dd, *J* = 14.4, 7.6 Hz, 1H), 1.34 (d, *J* = 7.2 Hz, 3H); LCMS rt 0.83 min, 100% purity at 214 nm; ESI-MS, *m/z* 271.0 [M-H].

((*S*)-2-amino-3-(3,4-difluorophenyl)propanoyl)-L-valine (12c). Prepared using Method B: ^{1}H NMR (400 MHz, D_2O) δ 7.32 – 7.16 (m, 2H), 7.07 (ddd, *J* = 8.3, 3.9, 1.7 Hz, 1H), 4.28 (t, *J* = 6.9 Hz, 1H), 4.02 (d, *J* = 6.2 Hz, 1H), 3.21 (qd, *J* = 14.3, 6.9 Hz, 2H), 2.14 – 1.97 (m, 1H), 0.91 (dd, *J* = 10.3, 6.8 Hz, 6H); LCMS rt 1.09 min, 100% purity at 214 nm; ESI-MS, *m/z* 299.1 [M-H].

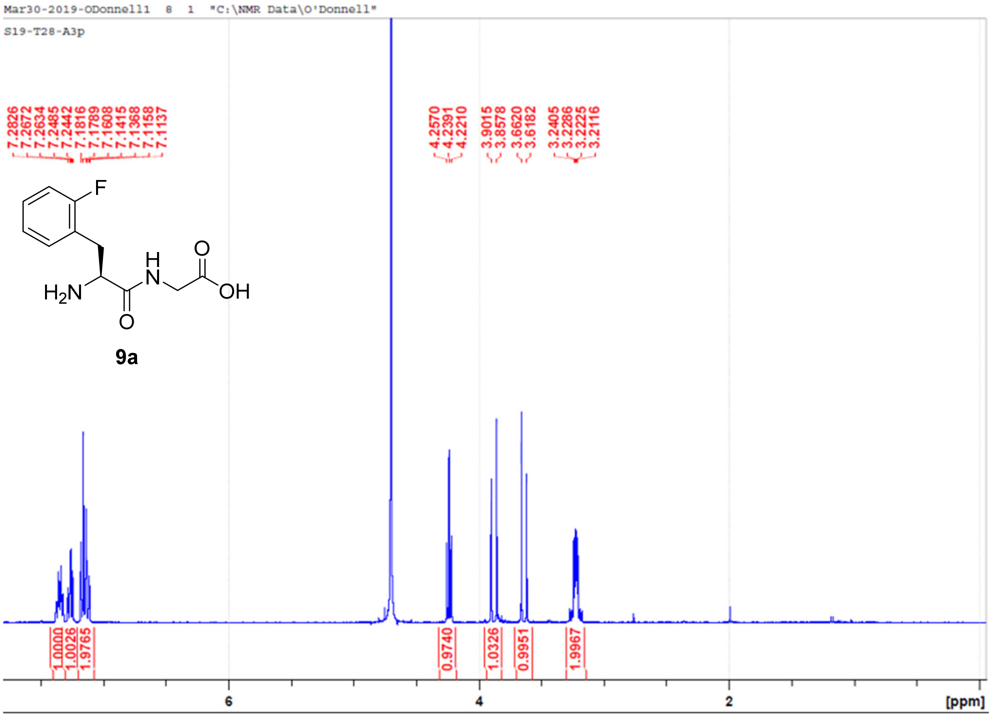

NoteBook Ref: S19-T28-A3

Method Name: QC_T0_HIGH.M
Operator: SYSTEM
Instrument Name: LCC_MS87_QCU3
Lilly Number:
Sample Postion: D1F-D1
Target Mass: 241
Comment:
Method Info: Waters X-Bridge C18 2.1x50mm 3.5u, @50C, 1.0mL/min, A:10mM Ammonium Bicarbonate pH 10; B: ACN; 0.2 min hold at 5%B, 5-100%B in 3.0min, hold 0.5min at 100%B.

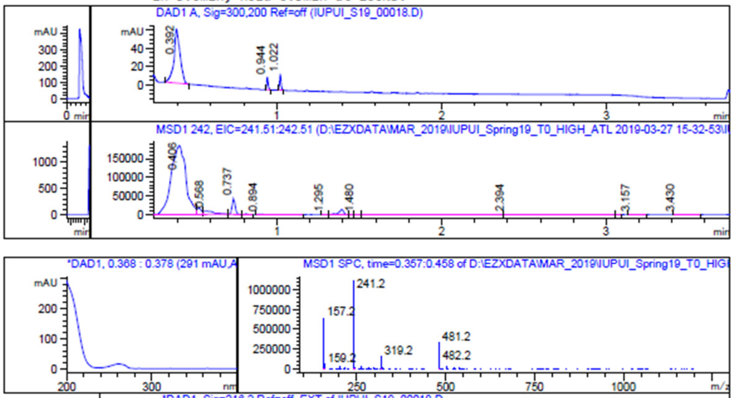

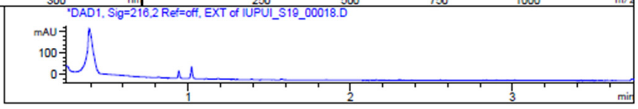

Results for peak at RT: 0.406min

Integration results for DAD1 A, Sig=300,200 Ref=off

Peak RT	Width	Height	% Area	MS Response (+)	MS Response (-)
0.39	0.03	59.2	88.0	241.2	239.2
0.94	0.01	13.9	5.3	296.0	312.0
1.02	0.01	16.9	6.7	329.2	310.0

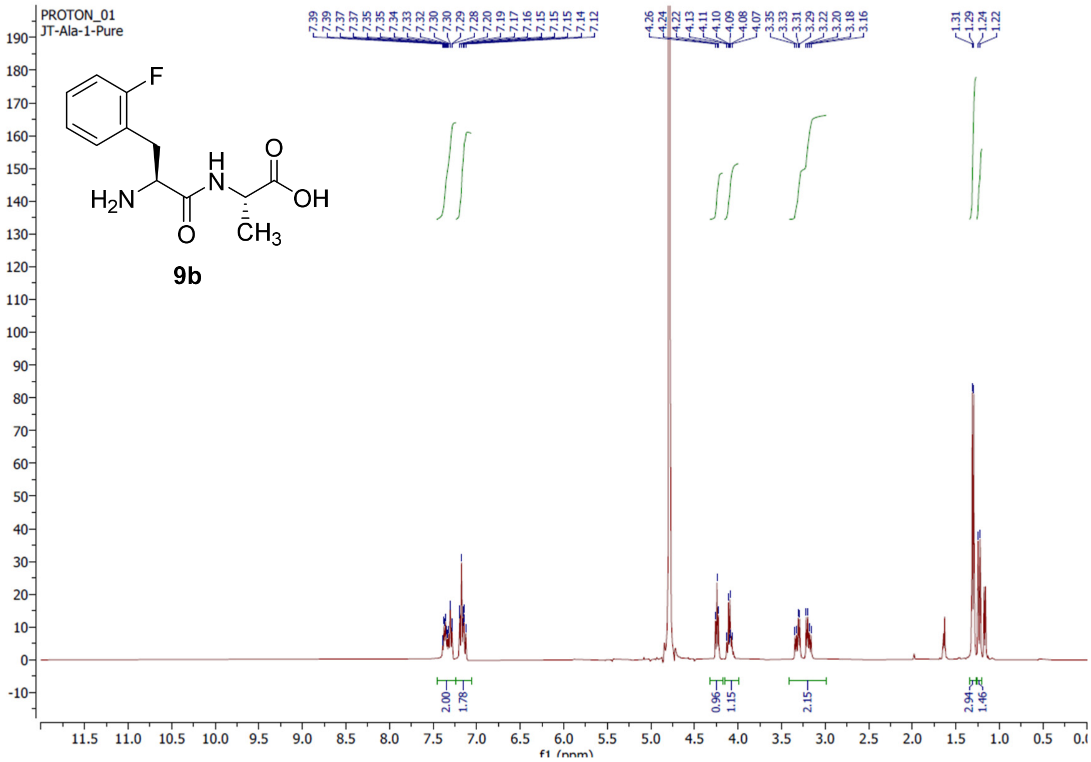

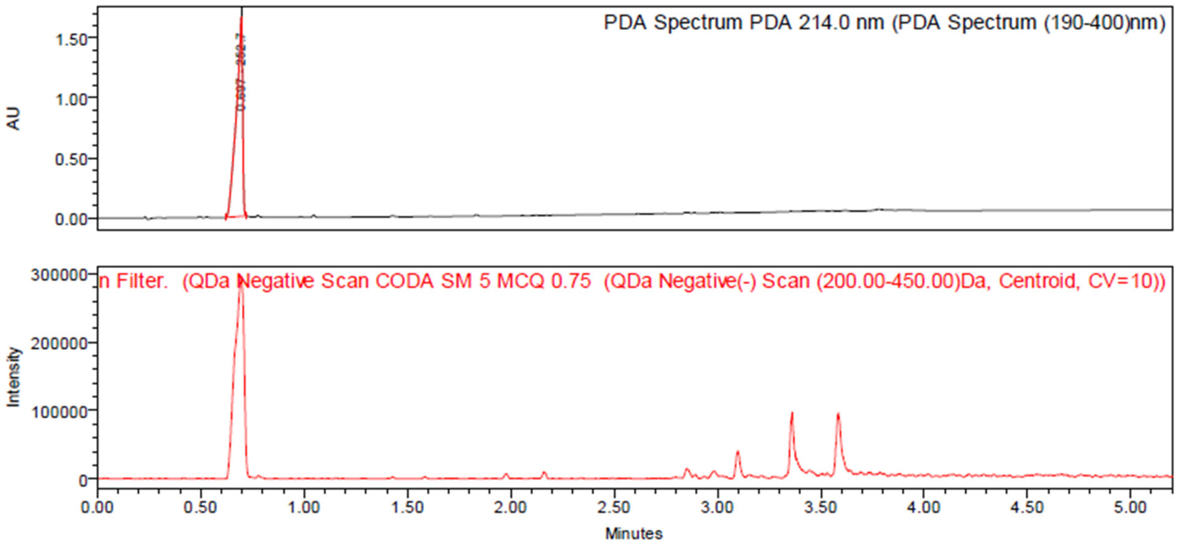

Peak Results

	RT	Area	Height	Base Peak (m/z)	% Area
1	0.697	3529883	1657296	252.69	100.00

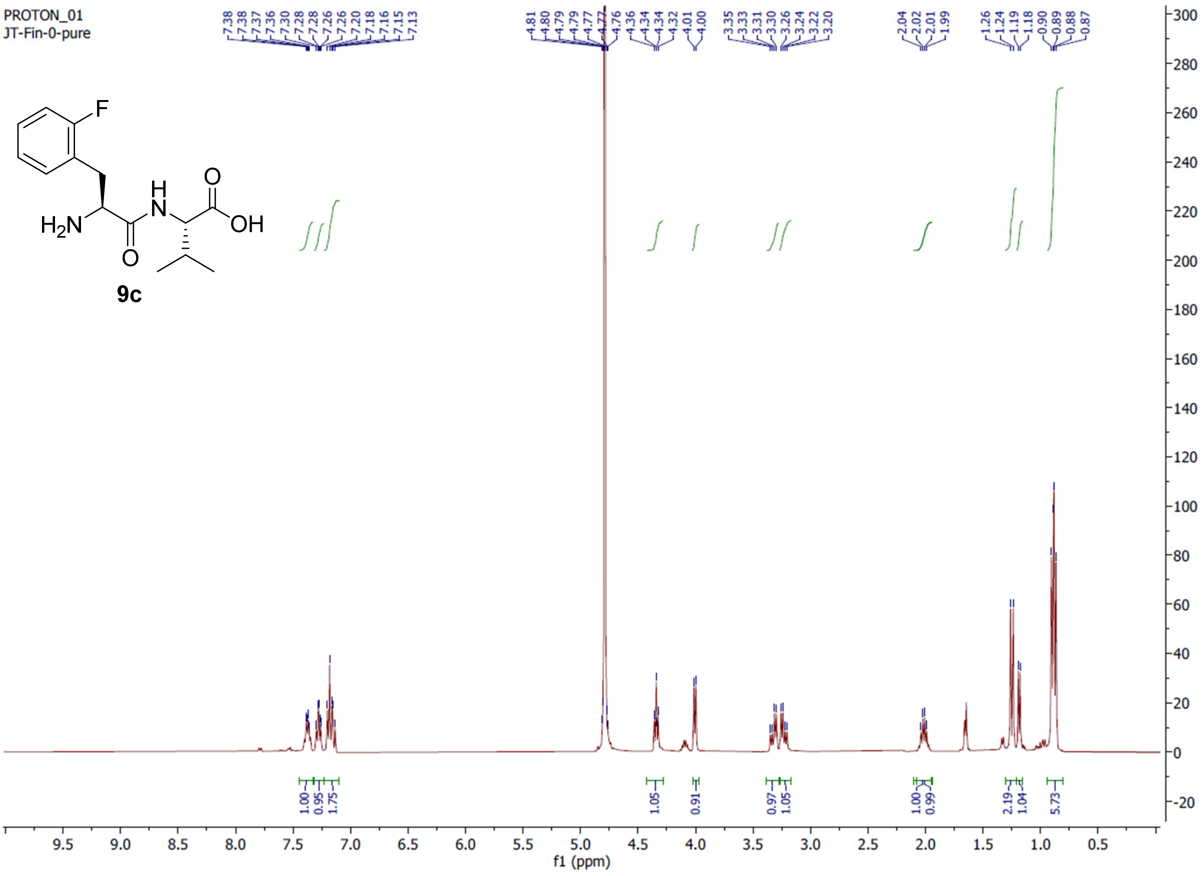

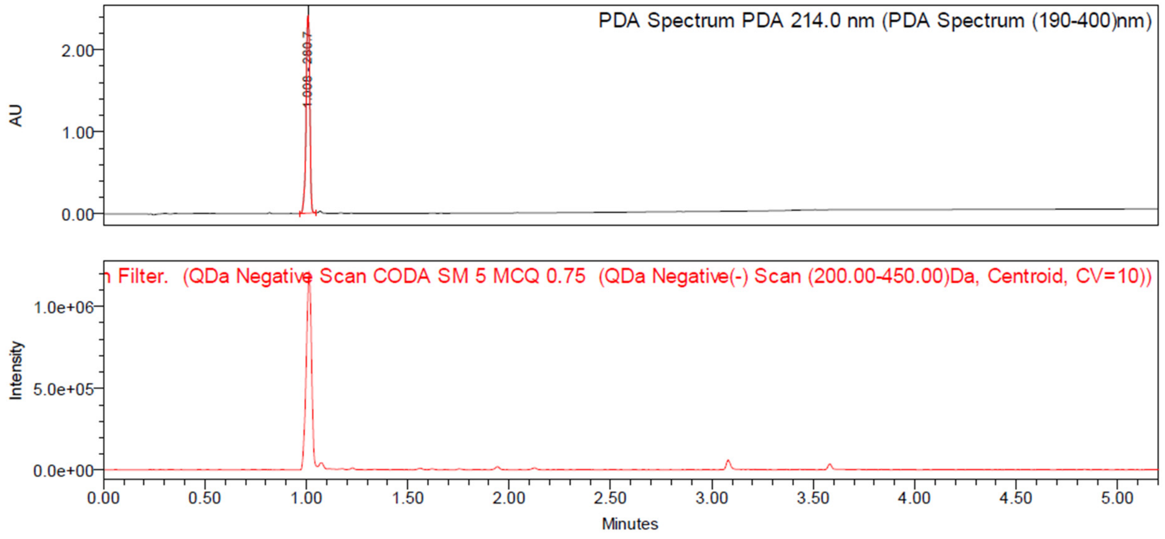

Peak Results

	RT	Area	Height	Base Peak (m/z)	% Area
1	1.008	3075893	2409202	280.72	100.00

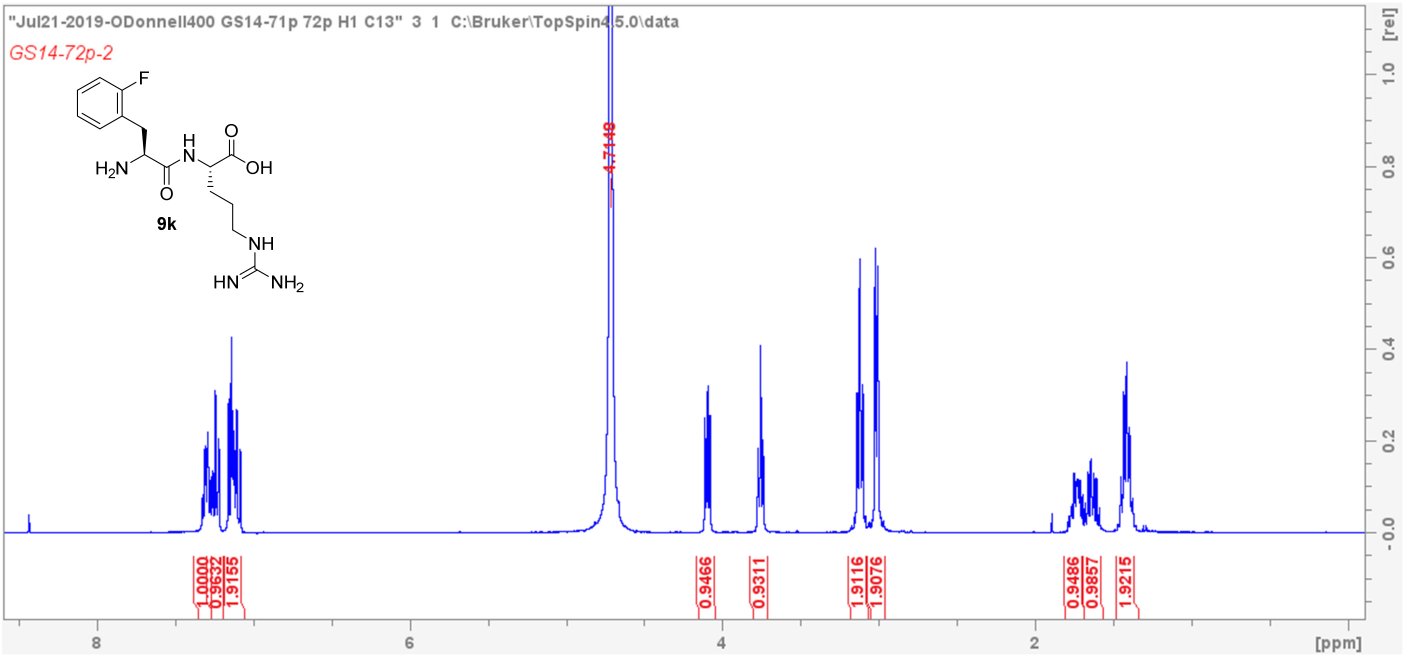


```
File D:\DATA\GENO\GS14-72.D                                 Tgt Mass (CHM):
Injection Date    : 19 Jul 19    1:39 pm -0500                    Seq. Line : 0
Sample Name       : GS14-72                                  Location  : Vial 61
Acq. Operator     : geno                                              Inj : 1
Spec. Reported    : MS Integration                            Inj Volume : 10 ul
Acq. Method       : D:\METHODS\GENO10GRADIENT.M
Analysis Method   : D:\METHODS\GENO10GRADIENTPURE.M
Sample Info :
Method Info :
```

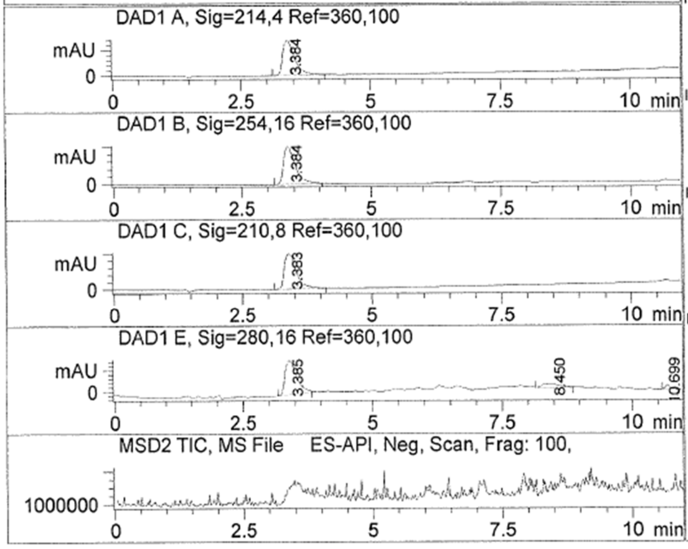

Integration Results for DAD1 A, Sig=214,4 Ref=360,100

RetTim	Width	Area	Height	Area%	MS(-)
3.38	0.25	5671.25	347.04	100.00	338.2

Integration Results for DAD1 B, Sig=254,16 Ref=360,100

RetTim	Width	Area	Height	Area%	MS(-)
3.38	0.24	398.51	24.79	100.00	338.2

Integration Results for DAD1 C, Sig=210,8 Ref=360,100

RetTim	Width	Area	Height	Area%	MS(-)
3.38	0.25	8183.45	499.24	100.00	338.2

Integration Results for DAD1 E, Sig=280,16 Ref=360,100

RetTim	Width	Area	Height	Area%	MS(-)
3.39	0.24	25.18	1.60	82.54	338.2
8.45	0.25	3.88	0.19	12.73	119.2
10.70	0.10	1.44	0.22	4.73	119.2

Integration Results for MSD2 TIC, MS File

RetTim	Width	Area	Height	Area%	MS(-)

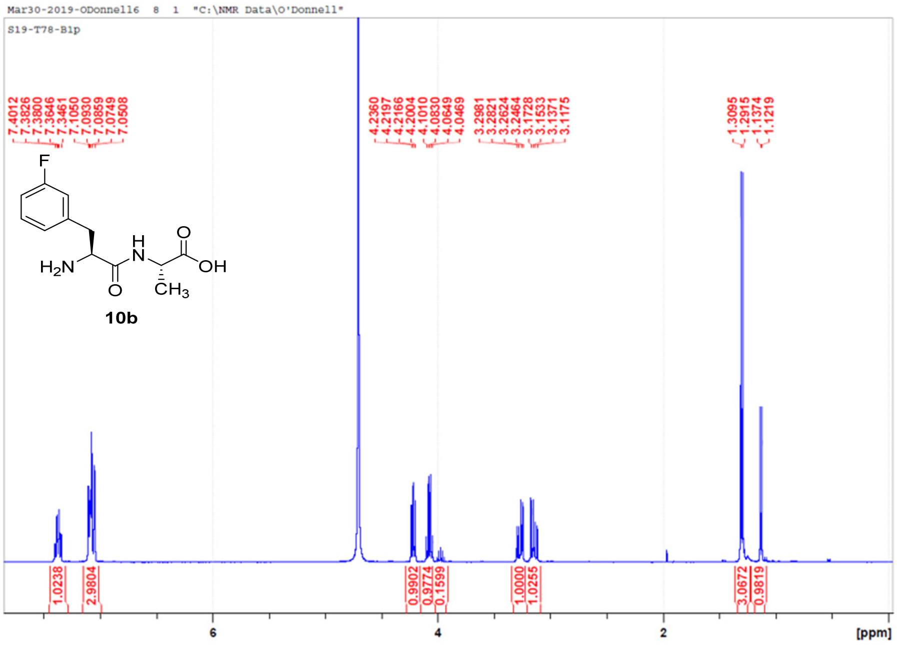

NoteBook Ref: S19-T78-B1

Method Name: QC_T0_HIGH.M
Operator: SYSTEM
Instrument Name: LCC_MS87_QCU3
Lilly Number:
Sample Postion: D2F-A4
Target Mass: 255
Comment:
Method Info: Waters X-Bridge C18 2.1x50mm 3.5u, @50C, 1.0mL/min, A:10mM Ammonium Bicarbonate pH 10; B: ACN; 0.2 min hold at 5%B, 5-100%B in 3.0min, hold 0.5min at 100%B.

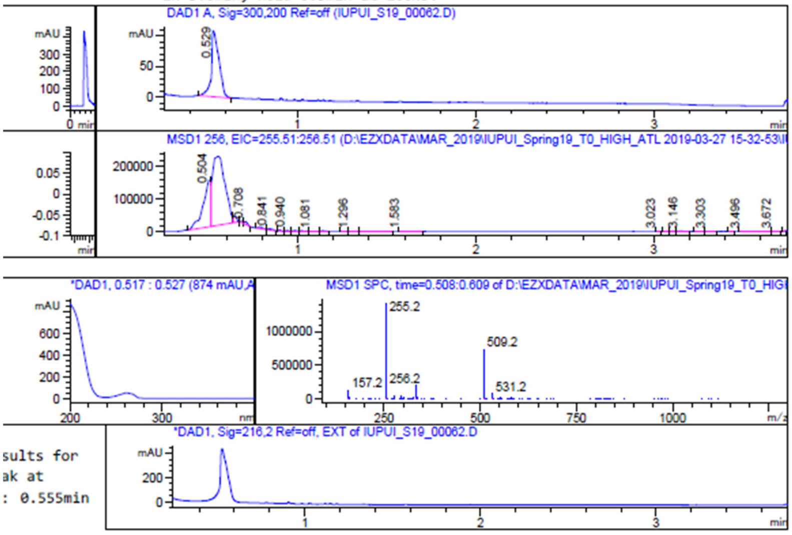

Integration results for DAD1 A, Sig=300,200 Ref=off

Peak RT	Width	Height	% Area	MS Response (+)	MS Response (-)
0.53	0.04	108.3	100.0	255.2	253.2

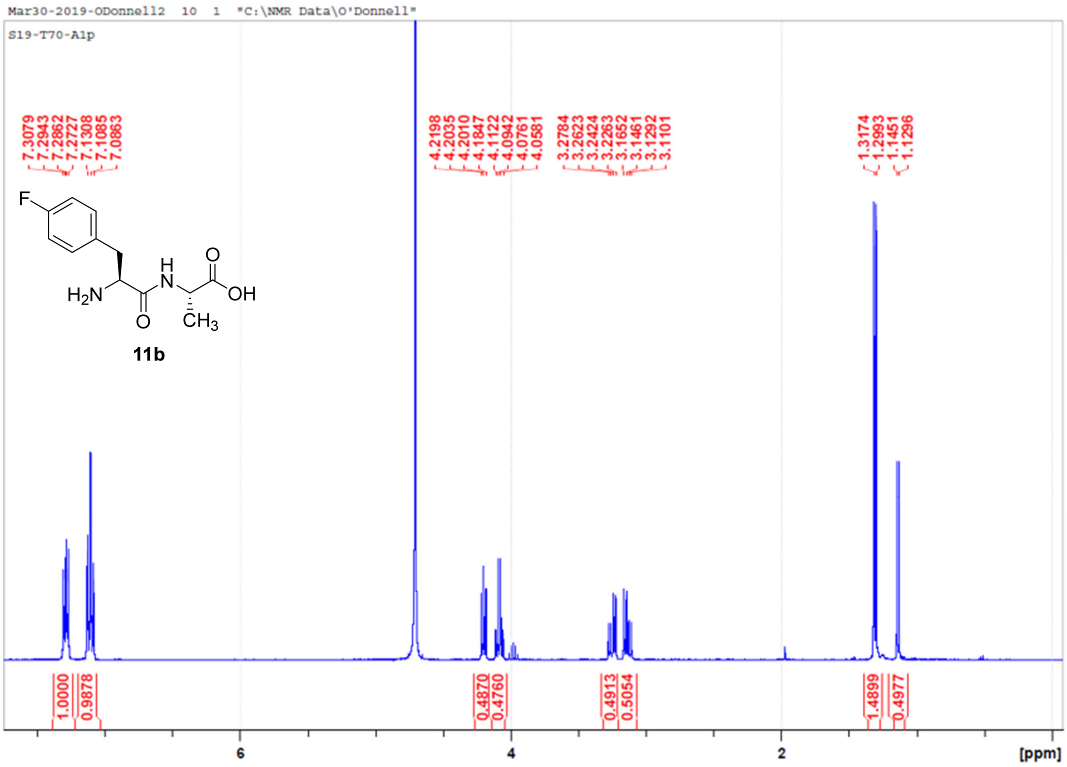

Sample Postion: D1B-A4 Target Mass: 255
Comment:
Method Info: Waters X-Bridge C18 2.1x50mm 3.5u, @50C, 1.0mL/min, A:10mM Ammonium Bicarbonate pH 10; B: ACN; 0.2 min hold at 5%B, 5-100%B in 3.0min, hold 0.5min at 100%B.

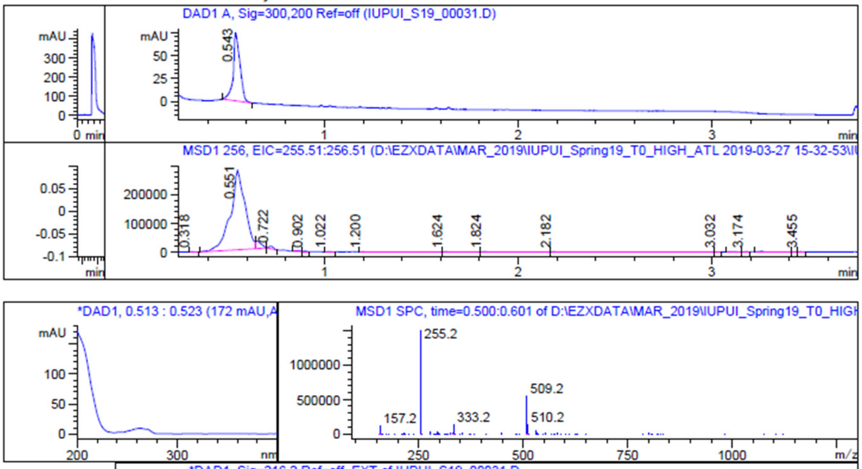

Results for peak at RT: 0.551min

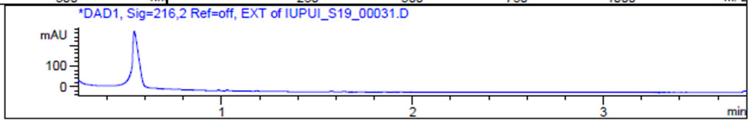

Integration results for DAD1 A, Sig=300,200 Ref=off

Peak RT	Width	Height	% Area	MS Response (+)	MS Response (-)
0.54	0.03	74.1	100.0	255.2	253.2

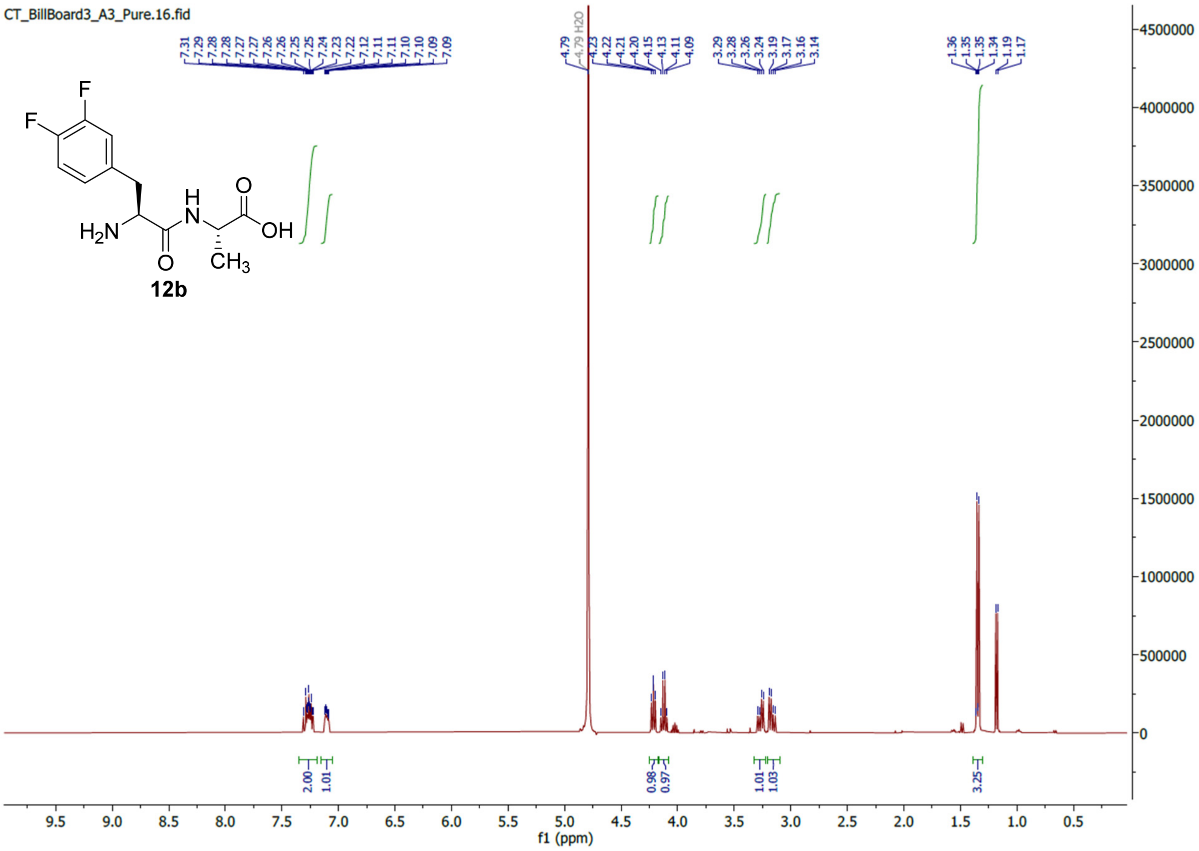

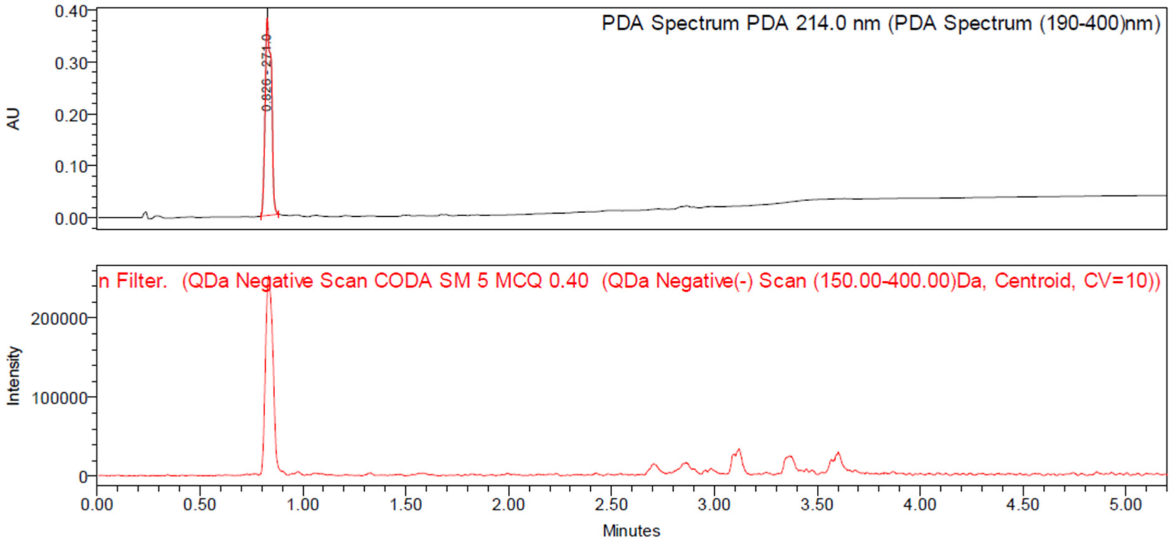

Peak Results

	RT	Area	Height	Base Peak (m/z)	% Area
1	0.826	798088	380428	271.03	100.00

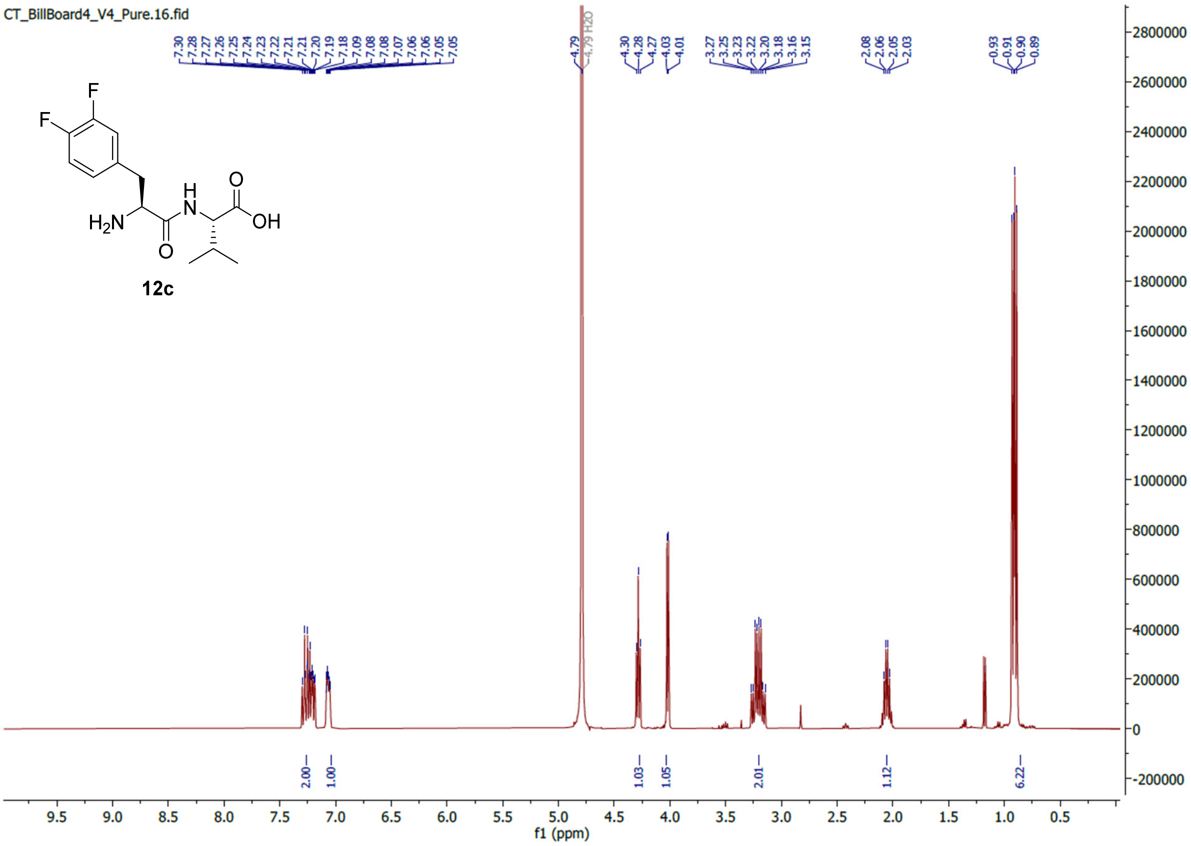

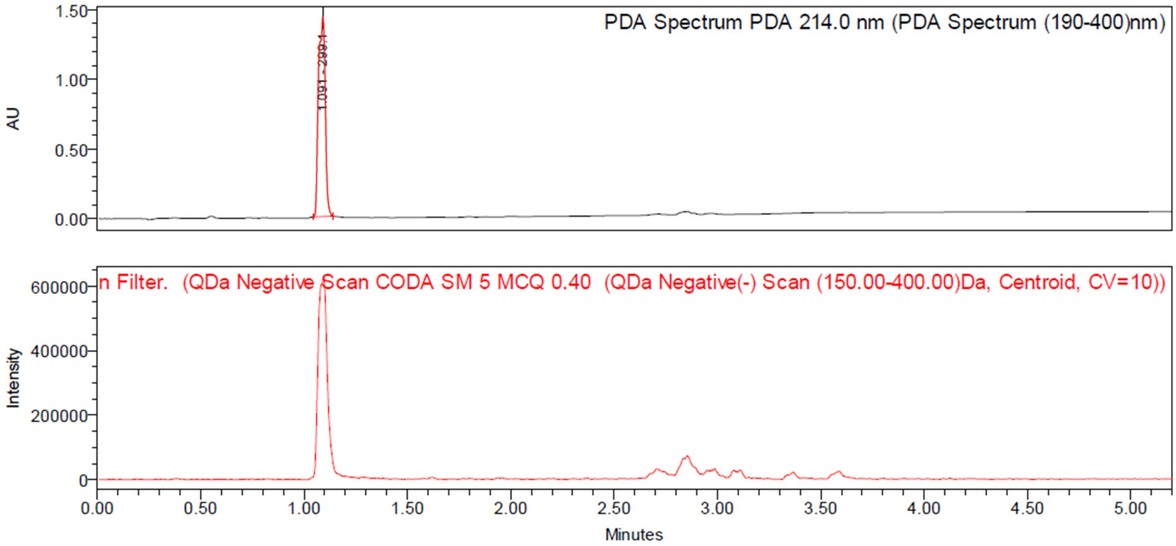

Peak Results

	RT	Area	Height	Base Peak (m/z)	% Area
1	1.091	3254476	1432066	299.06	100.00

Biological evaluation methods

Method A—biofilm growth measurement (IUI)

Biofilms were formed by a modification of the multiwell plate assay[3] with *Pseudomonas aeruginosa* strain PA14.[4] PA14 cultures were grown overnight in LB liquid medium at 37 °C with shaking. The cultured PA14 was then diluted 1:100 into M63 medium (15.1 mM $(NH_4)SO_4$, 40.2 mM K_2HPO_4, 22 mM KH_2PO_4) which had been completed with addition of 0.4% (w/v) arginine and 1mM magnesium sulfate (complete M63 media). Experimental compounds were evaluated at various concentrations in duplicate alongside 4-fluorophenylalanine (4FPhe, Millipore-Sigma, Burlington, MA) and/or tobramycin (Millipore-Sigma, Burlington, MA) in 24-well plates. Two test compounds at three different concentrations were placed in duplicate wells on one 24-well plate, and this was repeated by another biologist on a separate plate. Wells with bacteria alone and media alone were also included. The plates were covered and incubated at 37 °C for 24 h, statically. The liquid in the wells was then aspirated, the wells were washed with 2 × 1000 µL of water, aspirated and then treated with 750 µL each of a 0.1% crystal violet solution for 10 minutes. The stain solution was aspirated, and the wells were washed three times with 1000 µL of water, decanting each time. The plates were allowed to dry for 15 minutes, and the wells were then treated with 30% acetic acid. After 15 minutes the dissolved biofilm liquid was transferred to a 96-well plate and scanned spectrophotometrically for optical density at 550 nm.

The O.D. values were then entered into an Excel program. The four wells on both plates for each compound at a particular concentration were used to find the percent biofilm growth, which was determined by comparing the optical density of each well to the average optical density of the bacteria control wells. The average and standard deviations of the percent biofilm growth values were then determined in the Excel program. The Grubb's Test at a 95% confidence interval was used to determine any outliers. If any outliers were present, they were removed, and the mean and standard deviations were adjusted accordingly. This was repeated for all the duplicate wells on both plates, and the adjusted means and standard deviations of the percent biofilm growth values were reported.

Method B—bacterial and biofilm growth measurements (CC)

Biofilms were formed as described above (Method A), where overnight cultures of *P. aeruginosa* strain PA14 were diluted 1:1000 in complete M63 media and cultured in duplicate with experimental compounds or tobramycin. Stocks of these compounds were made in 10% (v/v) DMSO as the vehicle. Duplicate wells with untreated bacteria and media with vehicle alone were included as controls for maximum growth and background absorbance, respectively. After 24h incubation at 37°C, absorbance at 600 nm was measured on a Tecan Infinite 200 PRO plate reader to detect bacterial growth. Staining for biofilms using crystal violet was then performed as described above. To quantify biofilm formation, absorbance at 600 nm of the dissolved, crystal-violet-stained biofilm liquid was measured on a Tecan Infinite 200 pro plate reader and analyzed as described in the relevant figure legend.

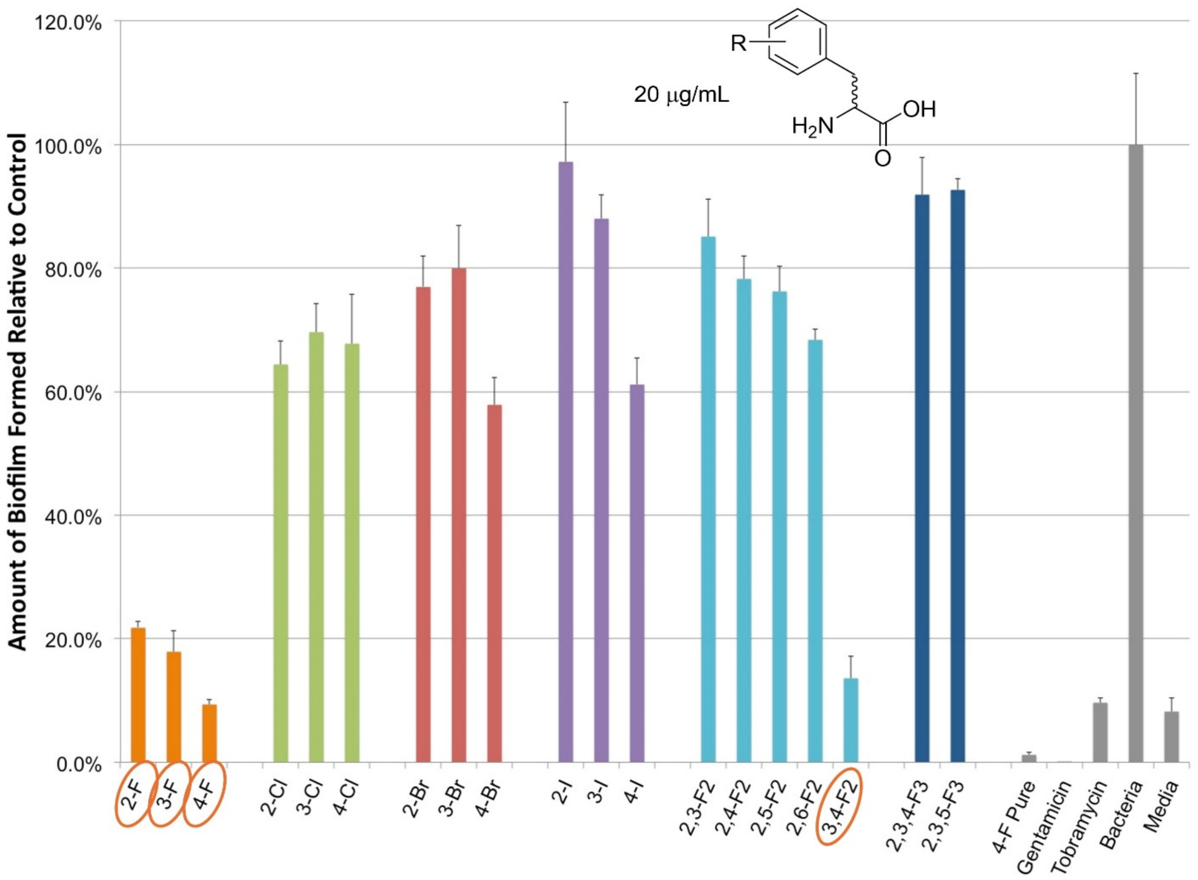

Figure S2. Comparison of biofilm formation of *P. aeruginosa* treated with 20 μg/mL racemic non-natural amino acid analogs (Method A).

Table S2. Representative dipeptide synthesis and *P. aeruginosa* biofilm formation measurements for **5-8**. Purity and identity of all compounds was confirmed by LC-MS. [a] Crude purity determined by LCMS; [b] Biofilm growth measured using Method A, and the average of biological replicates ± SEM are reported; [c] Biofilm formation measured at 10 µg/mL concentration using Method A; [d] Biofilm formation measured at 20 µg/mL concentration using Method A. Gold-highlighted cells indicate compounds that were fully characterized in Scott et al.[5]

entry	*N*-terminal amino acid (all L-amino acids)	*C*-terminal amino acid	% Crude purity range[a] (number of lots prepared)	Number of compound lots prepared	% purity of the compound lots tested in biofilm inhibition studies (number of compound lots tested)	% *P. aeruginosa* biofilm measured for 1 µg/mL compound[b] (number of biological replicates)
1	Ala	(*S*)-4-F-Phe	49-95	17	95, purified (2)	43 ± 10 (5)
2	Ala	(*R*)-4-F-Phe		1	purified (1)	102 ± 9 (2)
3	Ala	(*S*)-3-F-Phe	51-96	15	89-96, purified (6)	78 ± 6 (6)
4	Ala	(*R*)-3-F-Phe		1	Purified (1)	110 (1)
5	Ala	(*S*)-2-F-Phe	88-96	15	93-96 (4)	65 ± 15 (5)
6	Ala	(*R*)-2-F-Phe		1	Purified (1)	115 (1)
7	Ala	(*S*)-3,4-di-F-Phe	18-87	17	84, purified (2)	19 ± 16 (2)
8	Ala	(R/*S*)-4-F-Phe	23-97	92	Purified (7)	19 ± 5 (7)
9	Ala	(R/*S*)-3-F-Phe	55-93	44	Purified (4)	82 ± 3 (4) [c]
10	Ala	(R/*S*)-2-F-Phe	25-93	45	Purified (2)	76 ± 1 (2) [c]
11	Ala	(R/*S*)-3,4-di-F-Phe	44-90	32		28 [d]
12	Leu	(*S*)-4-F-Phe	42-86	2	42-86 (2)	53 ± 3 (2)
13	Leu	(*S*)-3-F-Phe	91-93	4	Purified (1)	71 (1)
14	Leu	(*S*)-2-F-Phe	92-95	2	*N/A*	*Not tested*
15	Leu	(*S*)-3,4-di-F-Phe	78-81	2	78-81 (2)	55 ± 13 (2)
16	Thr	(*S*)-4-F-Phe	86-92	4	86-92, purified (3)	42 ± 13 (5)
17	Thr	(*S*)-3-F-Phe	91-92	2	92 (1)	94 (1)
18	Thr	(*S*)-2-F-Phe	90-92	2	90-92 (2)	36 ± 7 (2)
19	Thr	(*S*)-3,4-di-F-Phe	83	2	83 (2)	80 ± 21 (2)
20	Val	*(S)*-4-FPhe	90-91	3	90-91, pure (3)	44 ± 13 (5)

21	Val	(*S*)-3-F-Phe	91-94	3	91-94, pure (3)	96 ± 8 (3)
22	Val	(*S*)-2-F-Phe	90-92	3	90-92, pure (3)	47 ± 17 (3)
23	Val	*(S)*-3,4-diFPhe	78-79	3	78-79, pure (3)	55 ± 29 (3)
24	Gly	*(S)*-4-FPhe	86-93	3	86-93 (3)	65 ± 8 (5)
25	Gly	(*S*)-3-F-Phe	91-94	2		
26	Gly	(*S*)-2-F-Phe	93-94	2		
27	Gly	*(S)*-3,4-diFPhe	80	2	80 (2)	72 ± 7 (2)
28	Lys	*(S)*-4-FPhe	92	1	92 (1)	65 (1)
29	Lys	(*S*)-3-F-Phe	53-86	2	53-86 (2)	91 ± 3 (2)
30	Lys	(*S*)-2-F-Phe	63-91	3	63-91 (3)	60 ± 14 (3)
31	Lys	*(S)*-3,4-diFPhe	87-88	2	87-88 (2)	100 ± 14 (2)
32	Ile	*(S)*-4-FPhe	85	1	85 (1)	43 (1)
33	Ile	(*S*)-3-F-Phe	88-90	2	88-90 (2)	86 ± 1 (2)
34	Ile	(*S*)-2-F-Phe	87-93	3	87-93 (3)	40 ± 12 (3)
35	Ile	*(S)*-3,4-diFPhe	79-81	2	79-81 (2)	95 ± 0.5 (2)
36	Met	*(S)*-4-FPhe	69	1	69 (1)	61 (1)
37	Met	(*S*)-3-F-Phe	70-71	2		
38	Met	(*S*)-2-F-Phe	77	2		
39	Met	*(S)*-3,4-diFPhe	59-77	2	59-77 (2)	55 ± 14 (2)
40	Trp	*(S)*-4-FPhe	29-71	2	29-71 (2)	97 ± 2 (4)
41	Trp	(*S*)-3-F-Phe	62-74	2	62-74 (2)	107 ± 8 (2)
42	Trp	(*S*)-2-F-Phe	74-75	2		
43	Trp	*(S)*-3,4-diFPhe	63-64	2	63-64 (2)	100 ± 10 (2)
44	Tyr	*(S)*-4-FPhe	94	2	94 (2)	64 ± 3 (4)
45	Tyr	(*S*)-3-F-Phe	92-94	2	92-94 (2)	74 ± 13 (2)
46	Tyr	(*S*)-2-F-Phe	65-93	2	65-93 (2)	71 ± 12 (2)
47	Tyr	*(S)*-3,4-diFPhe	90	2	90 (2)	74 ± 1 (2)
48	Ser	*(S)*-4-FPhe	93-94	2	93-94 (2)	67 ± 1 (3)
49	Ser	(*S*)-3-F-Phe	92-94	2	92-94 (2)	91 ± 14 (2)
50	Ser	(*S*)-2-F-Phe	94-95	2	94-95 (2)	65 ± 7 (2)

51	Ser	*(S)*-3,4-diFPhe	75-81	2	75-81 (2)	44 ± 6 (2)
52	Pro	*(S)*-4-FPhe	85-92	2	85-92 (2)	66 ± 3 (2)
53	Pro	(*S*)-3-F-Phe	87-90	2	87-90 (2)	100 ± 7 (2)
54	Pro	(*S*)-2-F-Phe	90-94	2	90-94 (2)	49 ± 3 (2)
55	Pro	*(S)*-3,4-diFPhe	79-86	2	79-86 (2)	53 ± 15 (2)
56	Asp	*(S)*-4-FPhe	85-86	2	85-89 (2)	66 ± 6 (2)
57	Asp	(*S*)-3-F-Phe	77-83	2	77-83 (2)	84 ± 2 (2)
58	Asp	(*S*)-2-F-Phe	88-91	2	88-91 (2)	91 ± 1 (2)
59	Asp	*(S)*-3,4-diFPhe	80-90	2	80-90 (2)	47 ± 5 (2)
60	Phe	*(S)*-4-FPhe	70-91	2	70-91 (2)	108 ± 4 (2)
61	Phe	(*S*)-3-F-Phe	91-92	2	91-92 (2)	85 ± 4 (2)
62	Phe	(*S*)-2-F-Phe	92-93	2	92-93 (2)	72 ± 4 (2)
63	Phe	*(S)*-3,4-diFPhe	66-84	2		
64	Glu	*(S)*-4-FPhe	59-91	2	59-91 (2)	106 ± 3 (2)
65	Glu	(*S*)-3-F-Phe	86-92	2	86-92 (2)	94 ± 2 (2)
66	Glu	(*S*)-2-F-Phe	86-90	2	86-90 (2)	79 ± 15 (2)
67	Glu	*(S)*-3,4-diFPhe	81	2	81 (1)	76 (1)

Table S3. Summary of dipeptide synthesis and *P. aeruginosa* biofilm growth for **9-12**. [a] School abbreviations: CC = Colorado College; GC = Goshen College; IUI = Indiana University, Indianapolis; SCU = Santa Clara University; UI = University of Indianapolis. [b] Biofilm formation measured using Method A, and the average and standard error of the mean were reported when there was more than one biological replicate.

Compound number	N-terminal amino acid	C-terminal amino acid	synthesis site(s) [a]	Number of compound lots prepared	Number of compound lots tested in preliminary screen	% *P. aeruginosa* biofilm formation at 1 µg/mL [b]	% *P. aeruginosa* biofilm formation at 5 µg/mL [b]
**9a**	2-F-Phe	Gly	IUI	1	1	55	4
**9b**	2-F-Phe	Ala	IUI	2	1	79	7
**9c**	2-F-Phe	Val	IUI, CC	3	3	29 ± 15	11 ± 5
**9d**	2-F-Phe	Leu	IUI	1	1	53	4
**9e**	2-F-Phe	Ile	CC	2	2	61 ± 5	3 ± 1
**9f**	2-F-Phe	Phe	*not prepared*				
**9g**	2-F-Phe	Tyr	CC, IUI	2	1	86 ± 5	34 ± 12
**9h**	2-F-Phe	Ser	IUI	1	1	56	6
**9i**	2-F-Phe	Thr	IUI	1	1	53	3
**9j**	2-F-Phe	His	IUI	1	1	98	85
**9k**	2-F-Phe	Arg	IUI	2	1	51 ± 45	12 ± 6
**9l**	2-F-Phe	Lys	*not prepared*				
**9m**	2-F-Phe	Met	IUI	1	1	93	55
**9n**	2-F-Phe	Asp	*not prepared*				
**9o**	2-F-Phe	Glu	IUI	2	2	74 ± 18	40 ± 35
**9p**	2-F-Phe	Gln	*not prepared*				
**9q**	2-F-Phe	Pro	IUI	1	1	99	101
**SI-9r**	2-F-Phe	Asn	IUI	1		*Not tested*	
**10a**	3-F-Phe	Gly	SCU	1	1	90	18
**10b**	3-F-Phe	Ala	IUI	1	1	70	3

**10c**	3-F-Phe	Val	CC, IUI	2	2	81 ± 2	17 ± 15
**10d**	3-F-Phe	Leu	SCU, CC	2	2	75 ± 28	3.3
**10e**	3-F-Phe	Ile	IUI	1	1	77	26
**10f**	3-F-Phe	Phe	IUI	1	1	85	50
**10g**	3-F-Phe	Tyr	IUI	1	1	91	57
**10h**	3-F-Phe	Ser	SCU	1	1	84	46
**10i**	3-F-Phe	Thr	IUI	1	1	87	46
**10j**	3-F-Phe	His	*not prepared*				
**10k**	3-F-Phe	Arg	SCU	1	1	100	95
**10l**	3-F-Phe	Lys	SCU	1	1	76	10
**10m**	3-F-Phe	Met	SCU	1	1	100	81
**10n**	3-F-Phe	Asp	IUI	1	1	105	57
**10o**	3-F-Phe	Glu	SCU	1	1	88 ± 7	76 ± 20
**10p**	3-F-Phe	Gln	UI	1	1	105	65
**10q**	3-F-Phe	Pro	SCU, CC	4	4	94 ± 3	45 ± 4
**11a**	4-F-Phe	Gly	SCU	1	1	22	1
**11b**	4-F-Phe	Ala	IUI, CC	4	4	14 ± 4	2.9 ± 0.5
**11c**	4-F-Phe	Val	CC	2	2	37 ± 25	2.6 ± 0.4
**11d**	4-F-Phe	Leu	SCU	1	1	59	2
**11e**	4-F-Phe	Ile	IUI	1	1	56	14
**11f**	4-F-Phe	Phe	IUI	1	1	66	42
**11g**	4-F-Phe	Tyr	IUI	1	1	89	1
**11h**	4-F-Phe	Ser	IUI, SCU	2	2	70 ± 19	51 ± 49
**11i**	4-F-Phe	Thr	IUI	1	1	38	1
**11j**	4-F-Phe	His	IUI	1	1	65	11
**11k**	4-F-Phe	Arg	SCU	1	1	97	72
**11l**	4-F-Phe	Lys	IUI, SCU	2	2	73 ± 5	7.5 ± 5.5
**11m**	4-F-Phe	Met	SCU	1	1	49	11

**11n**	4-F-Phe	Asp	IUI	1	1	96	3
**11o**	4-F-Phe	Glu	SCU	1	1	62 ± 12	1.7± 1.1
**11p**	4-F-Phe	Gln	UI	1	1	75	10
**11q**	4-F-Phe	Pro	SCU	1	1	82	2
**12a**	3,4-diF-Phe	Gly	SCU	1	1	23	1
**12b**	3,4-diF-Phe	Ala	IUI, CC	2	2	44 ± 8	6.2 ± 1.9
**12c**	3,4-diF-Phe	Val	IUI	1	1	46	8
**12d**	3,4-diF-Phe	Leu	SCU, CC	2	2	27 ± 18	3.3 ± 1.3
**12e**	3,4-diF-Phe	Ile	GC	2	2	78 ± 4	47 ± 0
**12f**	3,4-diF-Phe	Phe	GC	2	2	85 ± 1	60 ± 0.5
**12g**	3,4-diF-Phe	Tyr	*not prepared*				
**12h**	3,4-diF-Phe	Ser	SCU	1	1	69	7
**12i**	3,4-diF-Phe	Thr	IUI	1	1	61	13
**12j**	3,4-diF-Phe	His	*not prepared*				
**12k**	3,4-diF-Phe	Arg	IUI, SCU	3	1	86	18
**12l**	3,4-diF-Phe	Lys	SCU	1	1	90	14
**12m**	3,4-diF-Phe	Met	SCU	1	1	72	8
**12n**	3,4-diF-Phe	Asp	*not prepared*				
**12o**	3,4-diF-Phe	Glu	SCU	1	1	55 ± 12	13 ± 4
**12p**	3,4-diF-Phe	Gln	*not prepared*				
**12q**	3,4-diF-Phe	Pro	SCU	1	1	100	100

References

1. Dounay, A.B.; O'Donnell, M.J.; Samaritoni, J.G.; Popiolek, L.; Schirch, D.; Biernasiuk, A.; Malm, A.; Lamb, I.W.; Mudrack, K.; Rivera, D.G.; et al. Globally Distributed Drug Discovery of New Antibiotics: Design and Combinatorial Synthesis of Amino Acid Derivatives in the Organic Chemistry Laboratory. *J. Chem. Educ.* **2019**, *96*, 1731–1737. https://doi.org/10.1021/acs.jchemed.8b00942.
2. Tran, C.; Bergstrom, M.F.; Tee, J.; Brasuel, D.A.; Abbey, W.L.; Beizer, A.; Chua, A.E.; Diaz, A.R.; Grant, E.A.; Hernly, E.; et al. Greener Solid Phase Peptide Synthesis: Improved Procedure for Distributed Drug Discovery in Undergraduate Laboratories. *J. Chem. Educ.* **2026**, *103*, 1078–1085. https://doi.org/10.1021/acs.jchemed.5c01483.
3. O'Toole, G.A. Microtiter Dish Biofilm Formation Assay. *J. Vis. Exp.* **2011**, *47*, e2437. https://doi.org/10.3791/2437.
4. Liberati, N.T.; Urbach, J.M.; Miyata, S.; Lee, D.G.; Drenkard, E.; Wu, G.; Villanueva, J.; Wei, T.; Ausubel, F.M. An Ordered, Nonredundant Library of *Pseudomonas Aeruginosa* Strain PA14 Transposon Insertion Mutants. *Proc. Natl. Acad. Sci. USA* **2006**, *103*, 2833–2838. https://doi.org/10.1073/pnas.0511100103.
5. Scott, W.L.; O'Donnell, M.J.; Samaritoni, J.G. Antimicrobial Compounds and/or Modulators of Microbial Infections and Methods of Using the Same. U.S. Patent 11,453,699 B2, 27 September 2022..
